# Degradation and Protection of Materials from Cavitation Erosion: A Review

**DOI:** 10.3390/ma16052058

**Published:** 2023-03-02

**Authors:** Alicja Krystyna Krella

**Affiliations:** Institute of Fluid Flow Machinery PAS, Fiszera 14, 80-231 Gdańsk, Poland; akr@imp.gda.pl

**Keywords:** cavitation, cavitation erosion resistance, hardness, nitriding, boronising, shot peening, friction stir processing, PVD coating, HVOF coating

## Abstract

The phenomena of cavitation and cavitation erosion affect hydraulic machines, increasing their maintenance costs. Both these phenomena and also the methods of preventing the destruction of materials are presented. The compressive stress in the surface layer created from the implosion of cavitation bubbles depends on the aggressiveness of the cavitation, which in turn depends on the test device and test conditions, and also affects the erosion rate. Comparing the erosion rates of different materials tested using different tests devices, the correlation with material hardness was confirmed. However, no one simple correlation was obtained but rather several were achieved. This indicates that in addition to hardness, cavitation erosion resistance is also affected by other properties, such as ductility, fatigue strength and fracture toughness. Various methods such as plasma nitriding, shot peening, deep rolling and coating deposition used to increase resistance to cavitation erosion by increasing the hardness of the material surface are presented. It is shown that the improvement depends on the substrate, coating material and test conditions, but even using the same materials and test conditions large differences in the improvement can be sometimes gained. Moreover, sometimes a slight change in the manufacturing conditions of the protective layer or coating component can even contribute to a deterioration in resistance compared with the untreated material. Plasma nitriding can improve resistance by even 20 times, but in most cases, the improvement was about two-fold. Shot peening or friction stir processing can improve erosion resistance up to five times. However, such treatment introduces compressive stresses into the surface layer, which reduces corrosion resistance. Testing in a 3.5% NaCl solution showed a deterioration of resistance. Other effective treatments were laser treatment (an improvement from 1.15 times to about 7 times), the deposition of PVD coatings (an improvement of up to 40 times) and HVOF coatings or HVAF coatings (an improvement of up to 6.5 times). It is shown that the ratio of the coating hardness to the hardness of the substrate is also very important, and for a value greater than the threshold value, the improvement in resistance decreases. A thick, hard and brittle coating or alloyed layer may impair the resistance compared to the untreated substrate material.

## 1. Introduction

**Figure 1:**
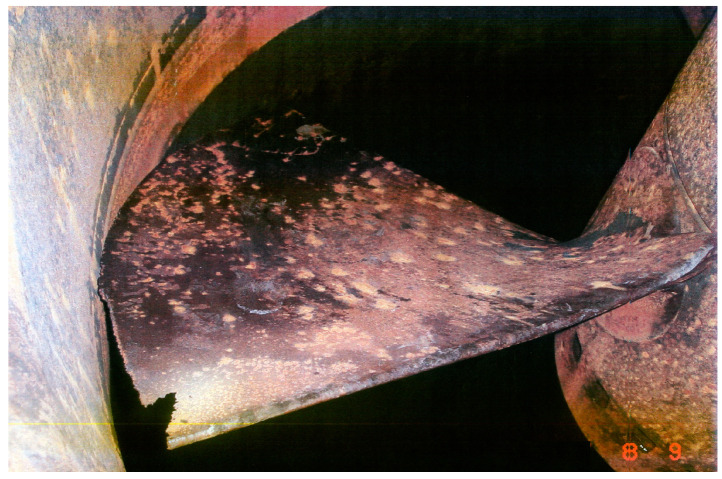
Damaged turbine blade caused by cavitation erosion (own resources).

Energy production together with environmental protection are currently the most urgent undertakings. The production of energy from renewable sources is especially recommended. Hydropower plants are the most common type of power plant, and they contribute 70% of the total energy production [1]. In addition, hydropower is currently one of the cheapest ways to generate energy. However, hydropower plants, especially hydro turbines, meet several problems, e.g., the degradation of turbine blades caused by erosion processes, namely, cavitation erosion, slurry erosion and a combination of these two processes. Slurry erosion concerns turbines working in the Himalayan region, China in Asia, and the Andes in South America, but cavitation erosion concerns all turbines regardless of where they work, as well as other devices, such as ship propellers. Figure 1 shows an eroded turbine blade. As it is seen, damage caused by cavitation erosion has the characteristic pattern of many pits of various sizes. 

Although in both phenomena material degradation occurs as a result of repeated dynamic loads over a small area, the failure mechanisms are different due to different velocities, as well as different sizes, shapes and properties of the impact loads. In slurry erosion, the component surface is exposed to the impact of solid particles; in cavitation erosion, micro-jets formed during the implosion of cavitation bubbles impact the surface of the material. For this reason, different standards are used for each phenomenon. The cavitation erosion test and test devices are described in ASTM G32 and ASTM G134. The slurry and solid particle erosion testing are described in ASTM G73. However, the terminology used to describe the erosion processes in both phenomena is in ASTM G40.

The requirements for increasing turbine efficiency are associated with an increase in rotational speed, which leads to an increased risk of initiation and development of cavitation as well as cavitation erosion. The latter leads to damage to the turbine structure. The consequence is a decrease in turbine efficiency and costly repairs. That is why it is so important to protect the turbine against cavitation erosion. As mentioned, other devices exposed to cavitation erosion are ship propellers and valves. 

There are two main ways to decrease the risk of occurrence of the cavitation phenomenon and damage caused by it: one is to design the turbine and turbine blades to minimize the risk of cavitation erosion, and the other is to select a resistant material. The best way is to use both these methods. To understand the problem of material degradation, investigations of the phenomenon of cavitation have been carried out. These investigations aimed to better know the stages of development of cavitation bubbles, including the implosion of cavitation bubbles. Simultaneously, experimental research on the properties affecting the resistance of materials to this type of damage has been carried out. There are several review papers [2,3,4,5,6,7,8,9] and books [10,11,12,13], but there is still the need to present and review the latest research referring to this problem: the degradation of materials caused by cavitation erosion. 

The aim of this paper is to present the state of the research on the phenomenon of cavitation erosion, as well as methods to protect the material from degradation. 

## 2. Cavitation and Cavitation Erosion Phenomena

Since the degradation of the material depends on the type of load to which it is subjected, it is advisable to present the phenomena of cavitation and cavitation erosion. The phenomenon of cavitation is described in detail in [10,11]. This section aims only to present the basic aspects related to the problem of material degradation. 

**Figure 2:**
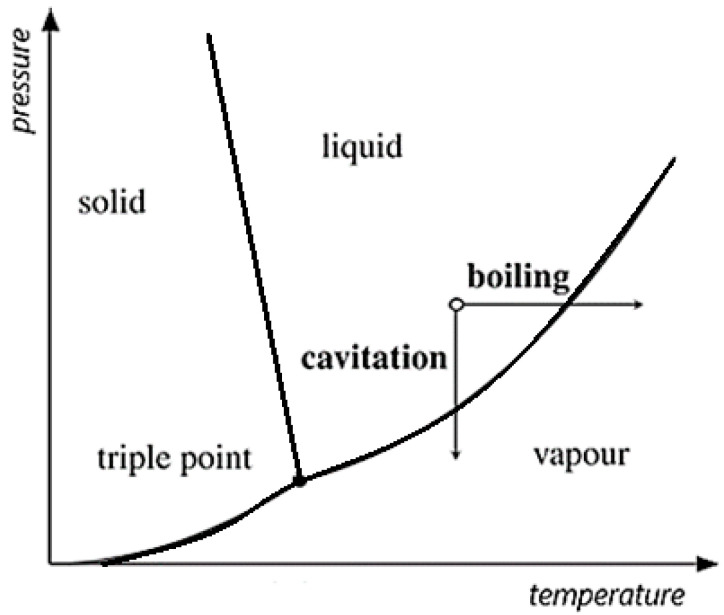
Scheme presenting the difference between cavitation and boiling.

Cavitation occurs in a liquid when the liquid pressure decreases below the vapor pressure without a change in the liquid temperature (Figure 2). This can be caused either due to fast-flowing liquid through any obstacle or due to the vibrating of an element in a liquid with high frequency. In the low-pressure area, bubbles develop from the nuclei, which may be undissolved air in the liquid. The nucleus is assumed to be spherical and to contain non-condensable gas of partial pressure Pg and vapor of the liquid of partial pressure Pv. Therefore, at the bubble’s surface, the balance among the internal pressure, the liquid pressure and surface tension can be written as follows:

P_L_ = Pv + Pg − 2γ/R, (1)

where P_L_ is the pressure in the liquid, γ is the surface tension parameter and R is the radius of the bubble. 

If the ambient pressure of the liquid changes very slowly, the radius of the bubble will change accordingly to accommodate the new equilibrium. This is accompanied by a modification of the pressure inside the bubble. Since cavitation bubble growth is rapid and gas diffusion occurs over a much longer time scale, the amount of gas inside the bubble remains almost constant. This causes the partial pressure of the gas to vary with the volume of the bubble. For a quasi-steady equilibrium, the gas follows an isothermal compression law, P_g_ is related to the initial values P_g0_ and R_0_ and to the new bubble radius R through the following:

P_g_ = P_g0_(R_0_/R)^3^. (2)

**Figure 3:**
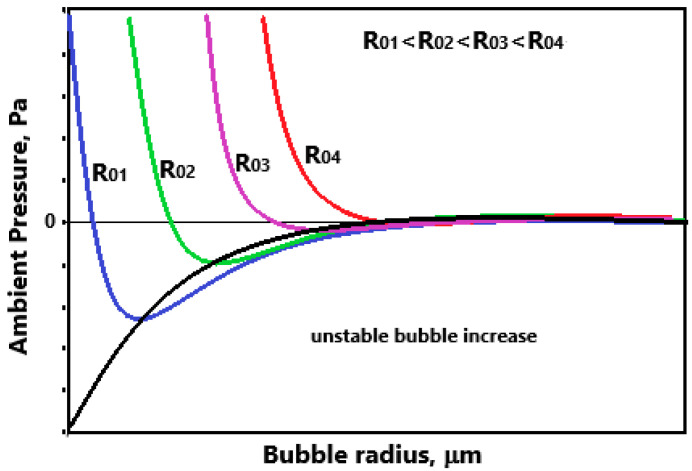
Correlation between pressure drop and bubble radius.

Figure 3 shows the correlation between pressure and bubble radius. Each curve has a minimum value below which there is no equilibrium bubble radius. If the pressure in the flow field drops below the critical pressure (the minimum value), an explosive bubble growth, i.e., cavitation, is created. Thus, a liquid flow experiences cavitation if the local pressure drops below the critical pressure, Pc. Figure 3 shows that the critical pressures are lower than the vapor pressure and depend on the initial bubble radius. Since the critical pressure varies with nucleus size, one requires knowledge of the nuclei size distribution in the liquid, which is essential information in the cavitation scaling study.

It should be added that the formation of bubbles in the liquid can also be caused by an increase in the temperature with unchanged pressure. The phenomenon of boiling occurs then, not cavitation. Figure 2 schematically shows the difference between these two phenomena.

As mentioned, cavitation occurs due to a rapid drop in the liquid pressure below the critical pressure. Thus, cavitation is the explosive growth of bubble nuclei to form cavitation bubbles which then collapse when exposed to large pressure variations. The bubble collapse is explosive, as is their growth, and is called an implosion. Practically, cavitation is identified as either the visual appearance of cavities in the absence of air injection and heat input or the acoustical emission of repeated high-pressure high-frequency recognizable sound produced by these cavities.

For a description of cavitation development, a cavitation number is used. According to the international standard ASTM G40, a cavitation number is a dimensionless number that shows the tendency for cavitation to occur in a flowing stream of liquid, and is computed from the following equation:

σ = (P_0_ − Pv)/(1/2 ρ v_0_^2^), (3)

where Pv is vapour pressure, Po is the static pressure in the stream in an undisturbed state, ρ is liquid density and v_0_ is undisturbed stream velocity. 

The bubble’s collapse is accompanied by compression of its content and the subsequent emission of a large amplitude pressure wave. If the bubble collapses close to a wall, a re-entrant jet is formed and crosses the bubble impacting the wall and creating high impulsive pressure. If the impulsive pressure resulting either from the impact of the micro-jet or from the impact of the shock waves exceeds some appropriate material threshold, such as the yield stress or ultimate strength, local damage is induced. This damage is called cavitation erosion.

The velocity of a micro-jet can vary in a wide range depending on the size of the imploding cavitation bubble and its gas content. Table 1 shows some values of micro-jet velocities. As it is seen, the micro-jet velocities are in the range of 30 m/s [14] to 877 m/s [15]. The presence of gases in the bubble has a damping effect and promotes re-regeneration of the bubble. It should be noted that the greater the liquid pressure drop, the smaller the nuclei which are activated as cavitation bubbles (Figure 3). The consequence of this is less and less gas content in the bubble and a higher speed of the micro-jet. 

Due to the high velocity of the micro-jet, the time of contact with the solid surface is very short: the order of a few µs, from 1.5 µs [27]–2 µs [23] to 10 µs [28]. Therefore, the impacts of micro-jets are called cavitation pulses. Multiple impacts of the pulses lead to an increase in residual stresses in the surface layer of the exposed material and the formation of pits and cracks, which join and cause the removal of surface material particles. Residual stresses are always compressive; their magnitude depends on the tested material, test device and intensity of cavitation (Table 2). 

There are two main types of test device: vibration and flow. The vibration test device is described in the international standard ASTM G32. For this reason and because of its simple design, it is the most popular. In this device, cavitation is generated by a horn vibrating with high frequency. Another stand that has been included in the ASTM standard is the cavitating jet stand (ASTM G134). Cavitation created by the cavitation jet is classified as flow cavitation. The concept of the cavitating jet described in the ASTM G134 standard was used to design other test devices. Some such devices are shown in [11]. Flow cavitation is also generated in cavitation tunnels. There are many cavitation tunnels, none of which have been included in the ASTM standard so far. The construction of tunnels is usually dependent on the capabilities and research needs of individual teams. Examples of cavitation tunnels can be found in [11,29]. Each test device has its range of cavitation intensity, which is due to the design. The said vibrating test device should work under strictly defined conditions (frequency 20 kHz and amplitude 50 µm), and the sample should be attached to the vibrating horn. However, the standard also allows other vibration conditions and the possibility of placing the sample in front of the horn, but this should be indicated in the test report or publication. In the case of flow devices, flow velocity has a key influence on the intensity of erosion [13,30,31].

**Table 2:** Cavitation-induced residual stresses.

Residual Stresses	Material	Test Device	Reference
−380 MPa	42 CrMo4 (AISI 4140)	Vibratory	[32]
−200 MPa	Ck45 (AISI 1045)	Vibratory	[32]
−550 MPa	316L	Cavitating jet in a pressurized chamber	[33]
−270 MPa	316L	Cavitating jet in water	[33]
−500 MPa	316L	Cavitating jet in air	[33]
−600 MPa	316L	Vibratory	[34]
−988 MPa	Ti-6Al-4V	Cavitation peening	[33]
−650 MPa	AISI 4140	Cavitation peening	[25]
−420 MPa	Ti-Ni alloy (Ti: 50.7%, Ni: 49.3%, vol. frac.)	Water jet impact	[35]

Table 2 shows that the residual stresses caused by cavitation can be as low as −988 MPa, which were obtained in the titanium alloy (Ti6Al4V alloy) exposed to cavitation peening [33]. However, in most cases, the compressive residual stresses are smaller. Mathias et al. showed that in the case of a vibratory test device working with the frequency of 20 kHz and amplitude of 40 µm, the cavitation-induced residual stress decreases to −380 MPa in 42 CrMo4 (AISI 4140) steel and only to −200 MPa in Ck45 (AISI 1045) steel after the same testing time [32]. Soyama showed that by testing the same material using different cavitation chambers, the residual stresses decrease to −270 MPa when the cavitating jet in water is used, or to −550 MPa when the cavitating jet in a pressurized chamber is used [9,33]. In addition, by testing the same material in the same test device, but in different conditions, the maximum comprehensive stresses are different. For example, in the case of 316L steel tested using cavitation peeling with p_1_ = 30 MPa and p_2_ = 0.1 MPa, the diameter of the nozzle throat affects the residual stresses and the thickness of the compressive layer. For the diameter of d = 0.35 mm, the residual stress at the surface was about −40 MPa and the thickness of the compressive layer was about 400 µm, while for d = 2 mm, the residual stress at the surface was about −270 MPa and the thickness of the compressive layer was about 1 mm. This increase in residual stresses was due to an increase in jet power [9,36,37]. In turn, the increase in the inlet pressure of the jet at a constant nozzle diameter and geometry resulted in a decrease in the residual compressive stresses and the thickness of the compressed layer [37].

**Figure 4:**
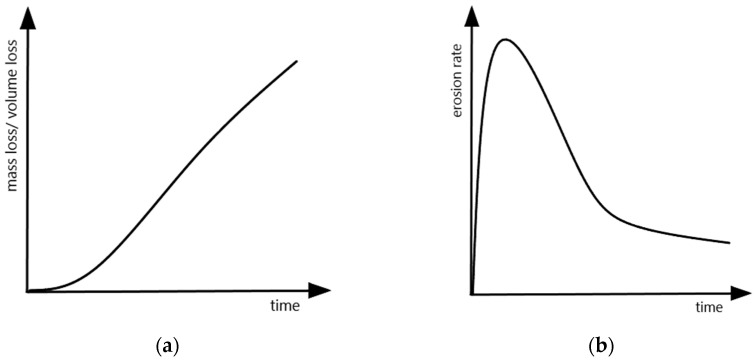
Cavitation curves: (**a**) mass loss/volume loss vs exposure time; (**b**) erosion rate vs exposure time.

The development of mass loss during cavitation erosion testing is typically represented by cavitation curves where one axis (x-axis) represents the duration of the test and the other axis (y-axis) represents the cumulative mass loss or volume loss. Very often, the erosion rate calculated as the ratio of mass loss (or volume loss) and exposure time is shown instead of mass loss or volume loss. The typical cavitation curves are shown in Figure 4. The use of mass loss or volume loss depends on the type of materials being tested and compared. Mass loss can be used for comparing materials with similar densities, whereas volume loss should be used for materials with dissimilar densities. In addition, there are other indicators when plotting cavitation curves, e.g., a mean depth of erosion (MDE) or mean depth of erosion rate (MDER). 

**Figure 5:**
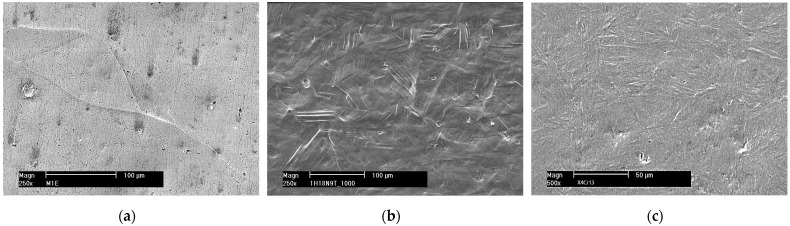
Cavitation erosion damage formed during the incubation period on the surface of (**a**) copper; (**b**) Cr18Ni9Ti austenitic stainless steel; (**c**) X4Cr13 martensitic steel (own investigations).

**Figure 6:**
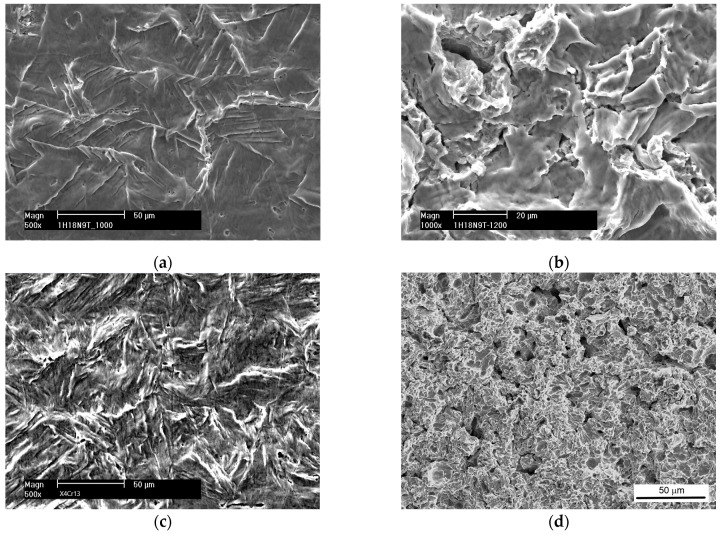
Cavitation erosion damage formed during the acceleration period on the surface of (**a**) Cr18Ni9Ti austenitic stainless steel—increase in slip bands density; (**b**) Cr18Ni9Ti austenitic stainless steel—formation of cracks and craters; (**c**) X4Cr13 martensitic steel—increase in surface undulation and material structure become more visible; (**d**) X4Cr13 martensitic steel—cracks and craters (own investigations).

There are typically three erosion periods: incubation, acceleration and deceleration. The incubation period is the initial stage of the erosion rate–time pattern during which the erosion rate is zero or negligible compared to later stages. During this period, tiny pits are created and grain boundaries become visible. In the case of austenitic steels, slip bands are also formed. Some examples of such damage are shown in Figure 5. The acceleration period is the stage following the incubation period during which the erosion rate increases from near zero to its maximum value. During this period, the number of pits increases and grain boundaries become more and more visible. The surface undulation also increases. In austenitic steels, the density of slip bands increases and deformation twins are formed. Craters and cracks develop. Some examples of such damage are shown in Figure 6.

The deceleration period is the stage following the acceleration period or the maximum rate period during which the erosion rate has an overall decreasing trend although fluctuations may be superimposed on it. The shape of the cavitation curve and the maximum erosion rate depend not only on the material tested but also on the intensity of cavitation erosion, which depends on the test device and test conditions. In turn, the test device and test conditions affect the residual stresses as shown in Table 2. There is therefore a correlation between residual stresses, the thickness of the compressed layer and mass loss (erosion rate) [36]. 

## 3. Cavitation Erosion of Solid Materials 

As shown in the previous section, cavitation and cavitation erosion are dynamic phenomena. Knowledge of the phases of material degradation and the factors influencing the rate of this degradation is important for prevention. Although there are many cavitation erosion testing devices, the most common is the vibrating device. The main advantages of this device are its inclusion in the international standard for testing resistance to cavitation erosion (ASTM G32) and its simple design. According to this standard, the vibration of the horn with a frequency of 20 kHz and an amplitude of 50 µm generates cavitation. However, the standard allows for other operating parameters, but then they should be provided in the description of the test results. Other research devices are cavitation tunnels, rotating disks and cavitating jet stands. A presentation of these test rigs and their descriptions can be found in [29]. Despite the ASTM G40 standard, the cavitation number (equation 3) is not given with cavitation erosion resistance tests. There are several reasons. Firstly, it cannot be used for all test devices. In the case of a vibrating device, the development of cavitation depends on the frequency and amplitude of vibrations. Secondly, the ASTM standard does not specify precisely the pressure and flow rate. According to the standard, these values should be values in undisturbed flow. In the case of flow cavitation and cavitation tunnels, it is not specified whether these values should be in front of or behind the cavitation-inducing obstacle. This can lead to a several-fold difference in the cavitation number. Therefore, it was accepted to provide only reliable terms and conditions. The development of cavitation in the vibrating devices is expressed by the frequency and amplitude of vibrations or by changing the standoff distance from the vibrating horn, and in the flow devices by the flow velocity and the shape and size of the obstacles or by the speed of the cavitation jet and the geometry of the nozzle in a cavitating jet device [7,35,36,38,39]. 

Since cavitation and cavitation erosion occur in liquids, mainly in water, very early research into material resistance involved stainless steels [40,41]. These studies showed that material structure and its hardness played an important role. The best resistance to cavitation erosion, which is the reciprocal of the erosion rate, was had by martensitic steels and the worst was had by ferritic steels, so the resistance improved with increasing steel hardness. However, investigations performed by Heathocock et al. [41] showed that 304 austenitic steel had comparable resistance to BS431S29 martensitic steel despite its lower hardness. A comparison of austenitic and various duplex steels confirmed the exceptional resistance of 304 steel to cavitation erosion [42]. This very good resistance was attributed to the biggest phase transformation of Feγ → Feα’. This transformation is related to the stacking fault energy (SFE) of the tested material and cavitation intensity. This phase transformation, i.e., its development and the impact of cavitation intensity on its occurrence during tests, was also shown in [38,40,43,44,45,46]. Research by Samtos et al. showed that the Feγ → Feα’ phase transition takes place mainly in the cavitation impulse interaction layer [43,47]. In A743 steel (CA6NM steel) tested with a vibrating device, which was in accordance with the ASTM G32-10 standard, this layer had a thickness of about 24 µm after 180 min of testing [43]. In the case of 304 steel, this layer had a thickness of 60 µm [47]. In addition, with increasing the testing time, the austenite fraction in the steel decreased up to some limit value. This threshold value depended on the steel grade. In the case of CA 6MN steel, the austenite fraction decreased from 16% to about 4%, and in the case of 304 steel, from about 95% to about 10%. With the increase in the phase transformation, i.e., the decrease in the austenite fraction, the hardness increased up to the saturation value. An increase in hardness with the testing time to the saturation value was also noted in [48]. Shin et al. [49] testing Fe–8Cr–0.7C–5Mn, Fe–16Cr–0.7C–5Mn and Fe– 16Cr–0.4C–5Mn steels noted that the steel with the lowest SFE (Fe–16Cr–0.4C–5Mn steel) had the best resistance due to the highest γ → α’ transformation during the cavitation erosion test and the highest volume fraction of transformed α’-martensite. The γ → α’ phase transformation was also attributed to a much better resistance of 18Mn18Cr0.5N steel than 0Cr13Ni6Mo steel and 0Cr16Ni5Mo steel [50]. Zhang et al. showed that along with the phase transformation, ηNi_3_Ti was precipitated in Cr-Mn-Ni steel during cavitation testing [45]. Both processes, phase transformation and precipitation, increase hardness and absorb some of the impact energy. 

As mentioned, very early investigations of bulk materials showed the impact of material hardness on erosion rate. Peng et al. [51] testing martensitic SUS630 stainless steel subjected to solution annealing at 1040 °C and aging annealing at 480 °C to 620 °C found that with increasing hardness, the yield stress and modulus of elasticity increased cavitation erosion resistance. In addition, these correlations were exponential. Hattori and Kitagawa conducted an extensive analysis of the cavitation erosion resistance of cast iron, carbon steel and non-ferrous metals [52]. They confirmed the exponential correlation between material hardness and resistance to cavitation erosion. For each material group, this dependence was as follows [52]:

ER = a HV^n^, (4)

where ER is erosion resistance, and a and n are the constants (different for each material group). For carbon steels, a = 5.8 × 10^−7^ and n = 2.4. For cast irons, these constants depend on the structure; for grey cast iron, a = 1.3 × 10^−5^ and n = 1.58, while for ductile cast iron, a = 2.1 × 10^−5^ and n = 1.61. For aluminum alloys, a = 0.75 × 10^−5^ and n = 1.6., and for copper alloys and titanium alloys, a = 2.8 × 10^−6^ and n = 2.19. They concluded that a different degradation mechanism affected erosive degradation in each group. In the case of cast iron, the structure and stress concentration around graphite have an influence on the strength, while in the case of aluminum alloys, the local overaging process is impacted, and in the case of copper and titanium alloys, the resistance to cavitation erosion depends mainly on the fatigue strength. 

The correlations obtained by Hattori and Kitagawa [52] led to a comparison of the erosion rates of different materials and their hardnesses. Based on the literature, such a comparison is presented in Table 3 and graphically in Figure 4. Table 3 shows that most of the investigations were carried out using a vibratory device. This indicates that, on the one hand, this device is the most advantageous for research, because it allows comparisons, and, on the other, the results obtained from other devices, especially those generating flow cavitation, which show the behavior of the material in other conditions, are very valuable. Carefully analyzing the data shown in Table 3, one can notice a large dispersion of erosion rates obtained for the same material tested using a vibratory device, i.e., the same device having with the same cavitation intensity. For example, the erosion rate of 304 steel can be in the range of 0.14 mm^3^/h [41] to 2.31 mm^3^/h [53]. The reasons may be differences in the properties of the steel, surface roughness and quality of the working fluid (e.g., content of undissolved air). This indicates the need to be very careful when using the results of others. 

**Figure 7:**
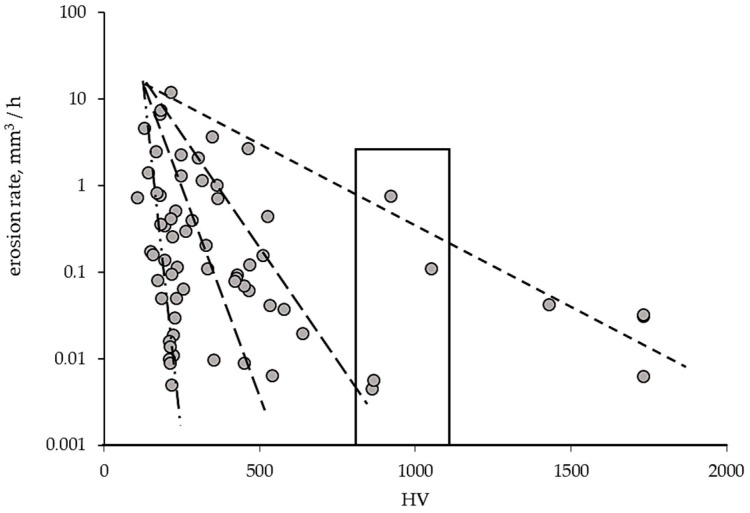
Correlation between hardness and erosion rate of various materials, based on Table 3.

Figure 7 confirms the relationship between the erosion rate and the hardness. However, not one but several such correlations have been revealed. The presence of several correlations is in accordance with Hattori and Kitagawa’s investigations [52]. Moreover, Figure 7 shows that different erosion rates can be obtained for the same hardness. For example, for the hardness in the range from 700 to 1100 HV, the erosion rates ranged from 0.001 to 1 mm^3^/h (marked in Figure 7). Thus, lower erosion rates were obtained for lower harnesses. This is because erosion rates of about 0.001 mm^3^/h are found in one relationship and erosion rates of about 1 mm^3^/h in another. Given that Figure 7 shows materials tested under different conditions, the presence of several correlations is likely related to the intensity of the cavitation load and the properties of the tested material, especially the response of the material to this type of dynamic load. This response depends on the structure as well as on the strength properties. It is known that ductile materials behave differently from brittle materials, e.g., steels compared to ceramics or porous materials. Moreover, ductile materials with FCC (Face Centered Cubic) structure, e.g., austenitic steels, behave differently under dynamic loads than materials with BCC (Body Centered Cubic) structure, e.g., ferritic stainless steels or carbon steels [68,69]. 

The very good correlation of the erosion rate with the hardness obtained in [52] motivated Hattori and Nakao to investigate further [48]. Investigations of carbon steels (S15C and S55C) subjected to different thermal treatments and nonferrous (Al and Cu) alloys did not prove the correlation shown in equation 4. Nevertheless, a good correlation of the rate of volume loss in the maximum rate stage with hardness and elastic modulus was obtained. According to [48], the following relation was noted:

VR ∝ HV^−3/2^ E^−2^, (5)

where VR is the volume loss rate, HV is Vickers hardness and E is Young’s modulus. Importantly, this correlation indicated that hardness alone does not affect the cavitation erosion resistance. Investigations performed by Peng et al., which examined SUS630 steel subjected to different thermal treatments to modify steel properties, confirmed the correlation of erosion rate with such material properties [51]. 

The effect of properties other than hardness on the cavitation erosion resistance have been investigated through tests of 13Cr–5Ni–Mo steel, Fe–Mn–Si–Cr shape memory alloys [38], S235JR carbon steel [39], C45 steel, a nodular cast iron (3.57 C, 2.51 Si, 0.23 Mn, 0.0044 P, Fe rest, wt.%) [70], and the rocks sandstone, shale and granite [71]. According to [38], both elongation and tensile strength have a higher influence on the resistance of the tested materials than hardness. The Fe-25Mn-6Si-7Cr-Cu shape memory alloy, which had a lower tensile strength, yield strength and elongation but a higher surface elasticity expressed as elastic depth after unloading in hardness measurements than the Fe–25Mn–6Si–9Cr–Cu shape memory alloy, was characterized by the best resistance to cavitation erosion. It was proven that increasing the surface elasticity decreases the erosion rate. Investigations of S235 JR carbon steel showed that, in addition to hardness, material properties such as elongation and impact energy obtained in the Charpy impact test influence the rate of erosion [39]. There was proposed a parameter, P, as follows [39]:

P = A^1.8^ × KV^2^/H^2^, (6)

where A is the elongation in%, KV is the impact energy obtained in the Charpy impact test in J and H is the hardness in MPa. With its increase, the erosion rate increases as well. In addition, heat treatment and the resulting changes in steel properties had little effect on the ratio of erosion rate to this parameter, regardless of the test conditions (cavitation intensity). Thus, the positive effect of elongation shown in [38] was confirmed. 

The tests of ceramics (alumina, zirconium oxide, alumina–zirconia composite and zirconium oxide–carbide composite) of very high hardnesses showed their good resistance to cavitation erosion [67]. However, no correlation with hardness was found. Alumina–zirconia composite, which had over five times better resistance than other ceramics, had the same hardness as other ceramics, comparable fracture toughness to zirconia and comparable Young’s modulus to alumina. In [67], it was concluded that the limitation of matrix grain growth during sintering together with the presence of compressive stresses in the matrix led to a significant improvement in cavitation wear resistance. Analyzing the results of testing rocks, a decrease in erosion rate with increasing yield strength can be also noted [71]. These studies showed that the structure of these rocks had a great impact on the resistance to cavitation erosion.

A limited effect of hardness on the cavitation resistance was also shown in the tests of C45 carbon steel and cast iron [70]. Despite having a similar hardness, the cavitation erosion rate of a nodular cast iron (3.57 C, 2.51 Si, 0.23 Mn, 0.0044 P, Fe rest, wt.%) was about 1.32 times higher than C 45 steel. The higher erosion rate of cast iron was attributed to the presence of stress concentrators around graphite in a metallic matrix. During the cavitation erosion test, the hard spheroidal graphites together with the surrounding area of ferrite were first removed [70]. Similar results were observed in [61,62,70,72]. This removal of graphite led to the formation of pits, which were stress concentrators and places of initiation and development of cracks in the soft and ductile matrix. A similar effect was also observed in other materials consisting of hard particles (precipitations) in a ductile matrix, e.g., in NAB (nickel aluminium bronze), AB (aluminium bronze) [64] and cobalt-based Stellates with Cr_7_C_3_ and Cr_3_C_2_ carbides [46,54]. 

Removal of hard phases from softer matrices leads to an increase in surface roughness (Ra, Rz and Rt parameters) [46,54,62,73]. The studies of cavitation erosion of rocks also confirmed the correlation between testing time and the roughness increase [71]. A similar relationship, but concerning ductile steel, was observed in [55,74,75]. The development of pitting was used by Fortes Patella et al. to calculate the volume erosion rate of copper, 316 stainless steel and aluminum [76]. Franc et al. [77] also confirmed a correlation between the pitting rate and flow velocity and a correlation between the pitting rate and material properties (elastic limit). Tzanakis et al. proposed a correlation for the prediction of volume loss based on roughness measurements in the following form [55]:

V/Vmax = a (Ra/Rmax)^n^, (7)

where V is the volume loss, Vmax is the maximum volume loss of the tested material, a is a constant, Ra is the Ra parameter and Rmax is the Ra parameter obtained for the maximum volume loss. 

Despite many proofs of the correlation of surface roughness or pitting with erosion rate, investigations performed by Chiu et al. [78] were in opposition to other investigations. The rate of roughness increase was different than the rate of erosion: at the beginning of the cavitation erosion test, the surface roughness (Ra parameter) increased rapidly and then its increase slowed down. This was explained by a change in the geometry of the pits. Moreover, in [31] a change in the geometry of the pits (depth, radius and volume of pits) was observed. In addition, the volume damage rate and pit number rate decreased with the testing time. This decrease may manifest itself as a decrease in surface roughness. 

The investigations of grain size confirmed an increase in cavitation erosion resistance with a decrease in grain size [79,80,81,82]. This increase was mainly caused by the improvement of properties such as hardness, tensile strength, yield strength and fatigue resistance. Studies of nitrogen austenitic stainless steel with an average grain size of approximately 2.5 µm and 37.5 µm showed that a 15-fold increase in grain size was accompanied by an approximately 65% increase in erosion rate, and damage developed mainly along the grain boundaries [79]. A similar effect of grain size was noted by Bregliozzi et al. [80]. In addition, it was found that the effect of grain size on the erosion rate can be intensified by the pH of the working liquid. In the case of 304 steel, an eight-fold increase in grain size caused an approximately 68%, 10% and 8% increase in the erosion rate for testing in a liquid with pH = five, seven and nine, respectively. In the case of a 16-fold increase in grain size, the erosion rate increased by about 106%, 25% and 13%, respectively. A different correlation was obtained by Lo et al. [82]: a bigger effect of grain size was observed in the test with distilled water than with 3.5% NaCl solution. This can be explained by the effect of grain size on corrosion resistance. As the grain size increases, the corrosion current density increases as well. Small grain sizes tend to increase passivation while slip bands that cause micro-steps on the surface of the tested material (increasing surface roughness) and promote pitting corrosion initiation sites are formed in large grains [82]. A similar effect of increasing passivation and increasing resistance to cavitation erosion (decreasing the rate of erosion) was obtained by adding Mo to 316 steel [83]. Steel with 8% Mo content was about five times more resistant to cavitation erosion than steel with 2.5% Mo content. 

## 4. Methods of Improvement of Cavitation Erosion Resistance

Since hardness plays a key role in resistance to cavitation erosion, and degradation concerns mainly the surface of eroded materials, to improve the resistance of materials to this type of degradation, various methods are used to increase the hardness of the material surface. The most often used were heat or thermo-chemical treatment (quenching or ageing heat treatments, surface hardening, carburizing and nitriding), machining (hot rolling and cold rolling), cladding (arc, laser and plasma), laser processing, coating deposition (PVD, CVD, thermal spaying, HVOF and HVAF) and others. Quenching or ageing heat treatments are applied to bulk metallic materials (inconel, stainless steels and aluminum alloys) to increase their hardness and strength properties. Surface hardening, carburizing, nitriding, hot or cold rolling, cladding, laser processing and coating deposition are used to modify the property of the material surface. Each method has its advantages and disadvantages. For example, coatings produced using cladding or laser processing are well bonded to the coating unlike adhesive coatings, but they have a heat-affected zone, which is unfavorable in terms of strength. On the other hand, the cladding of coatings of metastable austenite structure (MAS) allows the use of the martensitic phase transformation (γ → α’) initiated by cavitation loads. Korobov et al. showed that the TIG-deposited Fe-Cr-C-Al-Ti layer, which is MAS coating, had approx. 10 times better resistance to cavitation erosion compared to uncoated 316L steel [84]. This very good resistance was caused by the martensitic phase transformation (γ → α’). They showed that the martensite fraction in the MAS coating was over three times higher than in 316L steel. Thus, the phase transformation described in Section 3 applies to both the solid materials and the coating. In the case of coatings, its effect is intensified. The modification methods are described below. 

### 4.1. Thermo-Chemical Treatment 

The thermo-chemical treatment is the oldest method of improvement of properties of the surface layer, so also of cavitation erosion resistance. A summary of the impact of various treatments on the rate of erosion is presented in Table 4. 

Such treatments as carburizing and surface hardening are well-known methods of increasing surface hardness. Mitelea et al. [85] showed that carburizing of 16MnCr5 steel in gas at 880 °C reduced the rate of erosion by more than two times compared to this steel after only annealing at 670 °C. Induction hardening of carburized 16MnCr5 steel further reduced the erosion rate compared to this carburized steel. 

A comparison of the effect of plasma nitriding and salt bath nitrocarburizing on the cavitation erosion resistance of 13-4 CA6NM steel was studied in [88]. It was shown that a thicker hardened layer (190 µm) obtained in plasma nitriding improved cavitation erosion resistance by over 20 times compared to this untreated martensitic steel, while nitrocarburizing which formed a 120 µm thick hardened layer improved the cavitation erosion resistance by nearly four times. Although both processes allowed obtaining comparable maximum hardness in the uppermost region of the hardened layer, the difference in hardness profiles was the main factor affecting the improvement of resistance. The effect of plasma surface alloying with nitrogen and carbon was also investigated by Oliveira et al. [90]. Increasing the time of surface alloying from 3 h to 15 h increased the thickness of the alloyed layer, but there was no correlation for hardness: the lowest was for the alloying during 9 h and the highest was for 15 h. The resistance to cavitation erosion decreased with increasing surface hardness. Thus, the correlation opposite to Hattori and Kitagawa’s correlation (Equation (4)) was obtained. The main reason was increasing brittle fracture due to fatigue with increasing surface hardness. 

In addition to plasma nitriding, the effect of gas nitriding and laser gas nitriding on the cavitation erosion resistance was investigated. Li et al. [91] showed the effect of temperature (from 700 °C to 1000 °C) and time (from 4 h to 16 h) of gas nitriding on the structure and hardness of the hardened layer of commercial purity titanium (CP-Ti) and its cavitation erosion resistance. As nitriding temperature and time increased, the thickness and hardness of the modified layer increased. However, the hardest and thickest layer did not provide the best protection against cavitation erosion. A similar effect of high hardness leading to brittle fracture, as found by Oliveira et al. in [90], was obtained. Much better protective effects were obtained for titanium with the thinnest layer. The best resistance was obtained for titanium with the nitrided layer with the lowest hardness. Low resistance to cavitation erosion noted for the treatment at 850 and 1000 °C was caused by the defects (micro-cracks and pits) formed during the nitriding process. Polishing and removing the topmost defected layer of titanium nitrided at 850 °C for 8 h allowed for improving the resistance by about eight times. The effect of the high hardness of the nitrided layer was also shown in [92]. Although nitriding increased the resistance to cavitation erosion, greater mass losses were obtained for the alloy with a harder surface layer. In other words, a better improvement in resistance was obtained for the layer with a lower hardness. This is further proof that high surface hardness formed during nitriding does not always lead to improved resistance to cavitation erosion. 

Another process used to improve resistance is boronising [93]. The alloyed layer is built of three sub-layers: the FeB phase topmost sub-layer, the Fe_2_B phase sub-layer and the diffusion sub-layer. In [93], it was shown that the FeB sub-layer had the highest hardness. Extending the duration of the boronising process increased the thickness of the alloy layer (from 38.9 µm to 81.92 µm), especially for the FeB sublayer. The cavitation erosion tests showed that the lowest erosion rate had steel boronised for 2 h, and the highest erosion rate had steel boronised for 8 h with a thicker alloy layer because the borides undergo brittle fracture leading to detachment from the substrate. An increase in the thickness of the borides layer favors an increase in brittleness and the rate of erosion. 

The conducted review indicates that thermo-chemical treatment can improve the resistance to cavitation erosion by up to 20 times (Table 4). However, it should be taken into account that the high thickness of the brittle alloy layer may deteriorate the resistance compared to the untreated substrate material, as was the case with the stellite 250 (two-phase Co-Cr alloy) after low-temperature plasma carbonitriding at 380 °C for 15 h. 

### 4.2. Mechanical Treatment (Machining)

An increase in surface hardness as a result of mechanical treatment can be realized in many ways, e.g., shot peening, cold working (deep rolling) or friction stir processing. Table 5 shows a summary of the impact of various treatments on the rate of erosion.

Si et al. [65] used ultrasonic shot peening for increasing the hardness of the surface layer. With increasing the vibration intensity from 60% to 80% and/or peening duration from 60 s to 240 s, the compressive residual stress increased from 185 MPa to 332 MPa, resulting in an increase in surface hardness from 170 HV to 192 HV. Although this treatment improves by up to two times the resistance of the 2024T351 Al alloy compared to the untreated alloy, the increase in compressive stresses in the surface layer was not accompanied by a decrease in the rate of erosion. The best resistance to cavitation erosion was provided by peening at 80% vibration intensity and 60 s duration. This treatment resulted in a surface hardness of 175 HV and an increase in resistance of 2.14 times. The increase in shot peening time resulted in an increase in surface roughness (Ra parameter), which decreased the cavitation erosion resistance (increase in the erosion rate). Polishing the surface after shot peening decreased by about three times the erosion rate. On the other hand, it should be borne in mind that an increase in the stress level in the surface layer increases the risk of corrosion, especially when tests are carried out in corrosive solutions.

Muñoz-Cubillos et al. used deep rolling for increasing the hardness of the surface of 304 and 316 austenitic steels [94]. In the case of 304 steel, the surface hardness increased from 171 HV to 325 HV with a hardened layer thickness of 400 µm. This resulted in a decrease in the erosion rate by 2.23 times after 480 min of testing. In the case of 316 steel, the surface hardness increased to 270 HV (from 166 HV), the hardened layer thickness was 300 µm and there was a decrease in the erosion rate by 2.18 times after 480 min of testing. Thus, despite the differences in surface hardness, hardened layer thickness and martensitic phase transformation, the final improvement in cavitation erosion resistance was comparable. One of the reasons may be a phase transformation, which probably occurred during deep rolling, hence the large difference in the hardness and thickness of the hardened surface layer. 

Selvam et al. [95] noted that the friction stir process increases nearly 4–6 times the cavitation erosion resistance of 316 steel compared to the as-received alloy. This increase depended on the surface layer structure which in turn depended on the processing conditions. In the case of friction stir processing (FSP) performed at a rotational speed of 388 rpm, the grain size of the surface layer was 0.6 µm and the hardness was 420 HV. Using 1800 rpm, the grain was 0.9 µm and the hardness was 350 HV. Another consequence of this process was the formation of a martensitic phase: in the case of 1800 rpm, only a small 8% fraction of the martensite phase was formed, while at 388 rpm, the martensite fraction was as high as 45%. The cavitation erosion test carried out in water showed that FSP reduced the erosion rate by about five times for machining at 388 rpm and about 3.8 times at 1800 rpm. In the case of testing in 3.5% NaCl solution, the erosion rate decreased by about 4.5 times for machining at 388 rpm and about 3.4 times at 1800 rpm. The higher erosion rates obtained in NaCl solution were caused by corrosion processes favored by stresses introduced during FSP.

The effect of compressive stresses developed in the surface layer was also investigated by Qin et al. [63]. The compressive stresses of 60 MPa and 120 MPa were introduced using a self-made device. In opposition to the results shown in [65] and [95], the erosion rates increased. Similar to [95], this increase was higher in 3.5% NaCl solution due to corrosion processes. 

### 4.3. Laser Processing

The use of laser processing for the improvement of cavitation erosion resistance was widely investigated. Similar to the previous sections, a summary of the effect of laser processing on the erosion rate is shown in Table 6.

Early investigations carried out by Dube et al. [96] showed an improvement in the resistance to cavitation erosion. As expected, this improvement depended on the processing conditions. The best improvement was noted for the treatment producing the lowest surface hardness, while the worst was for the laser processing that produced the hardest surface layer. This confirms that surface hardness does not always improve resistance to cavitation erosion, which has been pointed out several times in this review. Similar results were found by Man el al. [97]. They noted that laser alloying using SiC and Si_3_N_4_ powders improved the resistance by up to three times compared to the untreated material. Laser melting did not significantly affect cavitation erosion resistance. However, not the hardness, but the content of Si_3_N_4_ powders affected the resistance. With the increase in the content of Si_3_N_4_ powders in the alloyed layer, the resistance to cavitation erosion increased. In addition, no correlation with corrosion resistance was found.

Wang et al. [54] who tested untreated 17-4PH steel, 17-4PH steel with laser cladding, Stellite 6B, wrought Stellite 6B and laser cladded C14 (cobalt-based alloy powders) found that the best resistance had laser cladded C14, which formed a layer with the highest surface hardness. They showed that the increase in surface roughness during the incubation period was similar to the volume loss in the later stage of erosion. The best resistance of laser cladded C14 specimen to cavitation erosion was attributed to the phase transformation of austenite to martensite that occurred during the cavitation test. As shown earlier, the phase transformation process promotes energy absorption and improves erosion resistance. Similar to Man’s investigations [97], no correlation with corrosion resistance was found. 

The effect of laser cladded coatings was also investigated by Jiang et al. [101], Yang et al. [102], Xu et al. [103] and others. Jiang et al. [101] studied the cavitation erosion behavior of Fe-Cr-Al-Ti-C-xY_2_O_3_ (x = 0, 0.2, 0.5 0.8 1.0 wt.%) laser coatings. The addition of Y_2_O_3_ improved the resistance, but the best resistance was obtained for the Y_2_O_3_ content of 0.2 wt.%. With the increase in Y_2_O_3_ content, the resistance to cavitation erosion decreased. 

The effect of laser power on the properties of the cladding on the improvement of resistance to cavitation erosion was studied by Yang et al. [102] and Xu et al. [103]. Yang et al. studied Ni-WC(-CeO_2_) cladding composites containing different CeO_2_ contents (0.9, 1.8 and 2.7 wt.%) and produced using different technologies [102], while Xu et al. tested WC-Ni coatings formed on 316 L stainless steel substrates under various laser operating conditions [103]. Yang et al. [101] noted that although laser power and CeO_2_ content affected hardness and the cavitation erosion resistance, no correlations were obtained. They attributed the improvement of resistance to cavitation erosion to the structure, in particular the morphology and arrangement of the WC phases and the eutectic Ni-WC networks in which the WC lamellae were arranged. Xu et al. [103] noted that the laser power affected the surface hardness and roughness, which in turn affected the resistance to cavitation erosion. Moreover, laser melting of the cold-sprayed WC-Ni coating reduced the erosion rate by more than 40 times. 

Another type of laser processing is laser shock peening. This treatment is used to refine the grain size and generate a high density of dislocation and compressive residual stress field in the surface layer. Zhang et al. [98] used it for the improvement of cavitation erosion resistance and tensile property welded ANSI 304 stainless steel. Laser shock peening increased the elastic limit and elastic modulus but decreased elongation. The change in steel property resulted in a three-fold increase in cavitation erosion resistance. Tong et al. [99] used laser shock peening with and without ablative coating. The cavitation erosion resistance increased by about 1.45 times for the processing with ablative coating and 2.13 times for the treatment without ablative coating in comparison to the untreated AA5083 aluminum alloy after the 300 min cavitation erosion test. Wang et al. [100] studied the effect of the number of coverage layers produced on the surface of 420 stainless steel during massive laser shock peening treatment on cavitation erosion and cavitation-silt erosion behavior. They noticed that the depth of the plastic deformation region after laser processing was about 36 µm for one coverage layer and about 54 µm for two coverage layers. Increasing the number of coverage layers increased the compressive residual stresses and surface hardness. The surface hardness increased by 1.14 and 1.22 times compared to untreated steel which was accompanied by an increase in cavitation erosion resistance by 1.5 and 1.8 times, respectively. Since no phase transformation of martensite (α′) into austenite (γ) induced by laser treatment was observed, the improvement resulted from the increase in hardness and the generation of a compressive stress field in the surface layer. 

### 4.4. PVD Coating Deposition

PVD (Physical Vapor Deposition) coatings are thin and hard coatings used mainly in tribological applications because of their low friction coefficient. Besides wear resistance, the deposition of PVD coatings can improve also fatigue and cavitation resistance [104]. The improvement depends on the coating properties, especially hardness, H, elastic modulus E, H/E or H^3^/E^2^ ratios, and adhesion. Despite the high hardness of PVD coatings, cavitation erosion tests showed their micro-undulation in the initial period of degradation. This micro-undulation effect was the result of the coating conforming to the undulated substrate. A summary of the effect of the deposition of PVD coating on the cavitation erosion rate and resistance is shown in Table 7.

Godoy et al. [89] investigated the effect of deposition Cr_1-x_N_x_ coating on plasma nitrided and non-nitrided 1045 carbon steel. As shown in the earlier section, plasma nitriding increases surface hardness and in most cases improves the cavitation erosion resistance (Table 4). Godoy et al. [89] assumed that depositing a hard coating on a hard substrate improves the resistance to cavitation erosion. The tests carried out confirmed this assumption; resistance increased by up to 7.5 times. It should be added that deposition of this coating on the untreated substrate also improved resistance: the rate of erosion was reduced by two times. The positive effect of deposition of Cr-N coating was also obtained in [105]. It was shown that the improvement in cavitation resistance increased with increasing the coating and substrate hardness. The best improvement achieved was 2.7-fold, so lower than that obtained by Godoy et al. [89]. A much better improvement of the cavitation erosion resistance by deposition of AlTiN and TiAlN coatings on the 304 substrates was shown in [107]. The magnetron-sputtered AlTiN and TiAlN coatings increased the resistance up to 11 times and 6.6 times, respectively. The better protection property of AlTiN coating was attributed to higher hardness, adhesion, H/E and H^3^/E^2^ ratio. Another widely studied PVD coating is the TiN coating. However, the improvement in resistance to cavitation erosion was lower than in the case of Cr-N coatings [110]. On the other hand, Liu et al. [108] showed that the deposition of the TiN coating had a slight effect on reducing the rate of erosion compared to the uncoated 304L steel. Ma et al. [106], examining a commercially produced TiN coating on Ti6AlV alloy, showed a decrease in resistance to cavitation erosion compared to the uncoated substrate. These results confirm the need for caution in not concluding too quickly after one or two works.

The tests of NiCrAlTi and NiCrAlTiN coatings with different nitrogen contents showed the best protective properties of the NiCrAlTi coating: the coating without the nitrogen addition [108]. The cavitation erosion resistance increased by 49 times due to the deposition of NiCrAlTi coating on the surface of 304 steel substrates. In the case of coatings with the addition of nitrogen, their erosion rates were lower than uncoated steel (so the resistance increased). However, the erosion rate increased with increasing N_2_ flux during coating deposition, and thus the resistance decreased compared to the NiCrAlTi coating without nitrogen addition. Deposition of NiCrAlTiN coatings improved the resistance from 1.8 times to 25 times. 

A comparison of single and multilayer coatings of the same thickness showed the better resistance of multilayer coatings [106,109,111,112]. The structure of multilayer coatings plays a significant role in the properties and cavitation erosion resistance. Momeni et al. [109] noticed that as the thickness of the upper hard TiCN layer increased, the hardness and resistance to cavitation erosion increased as well. They did not note the correlation between adhesion and cavitation erosion resistance, so they concluded that good coating adhesion does not guarantee improved resistance. Ma et al. [106] also confirmed the better resistance of multilayer CrAlYN/CrN coatings deposited on the Ti6AlV substrate compared to TiN coating. The resistance of the CrAlYN/CrN coating deposited with low ion bombarding energy was 13.5 times better than the uncoated substrate. Damage in both coatings was induced by surface defects created during the coating deposition process. However, in the case of the CrAlYN/CrN coating deposited with high ion bombardment energy, cracks were mainly generated in the substrate leading to the removal of the coating. In the case of the CrAlYN/CrN coating deposited with low ion bombardment energy, cracks appeared along the columnar coating grains. This can be correlated to the denser atomic packing of the {111} crystallographic planes in the coating deposited with high ion bombardment energy, which delay crack initiation in the substrate more effectively as compared to the lower atomic density {200} planes of the coating deposited with low ion bombardment energy. 

**Figure 8:**
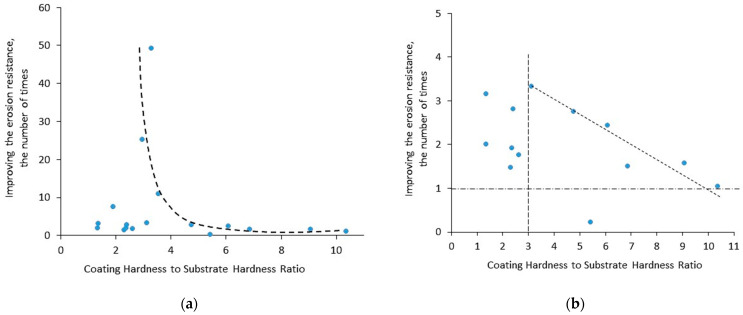
Correlation between the hardness ratio and improvement in erosion resistance of various materials, based on Table 7. (**a**) All data are included in Table 7; (**b**) Enlarged part of Figure 8a for the improvement of the resistance by up to five times.

Analyzing Table 7, one can notice that a large difference between the coating and substrate hardness (large ratio of coating hardness to substrate hardness) may lead to the reduction of the erosion rate. For example, the deposition of TiN coating with a hardness of 2200 HK on Ti6AlV substrate with a hardness of 405 HK caused an increase in the erosion rate (decrease in cavitation erosion resistance) [106]. Thus, in the case of adhesive coatings (PVD coatings are such coatings), a high ratio of coating hardness to the substrate may result in a deterioration of resistance, not its improvement. Based on Table 7, the effect of the ratio of the coating hardness to the hardness of the substrate on the improvement of cavitation erosion resistance has been developed and is shown in Figure 8. The greatest improvement in erosion resistance occurred for such a ratio of about three (Figure 8a). With a further increase in this ratio value, the improvement in resistance decreased (Figure 8b). For this ratio of less than 3, the dispersion of the erosion resistance improvement is quite large, from about 1.2 to 3.2 times. This dispersion may be affected by test conditions and other coating and substrate properties. The high hardness of the coating and the high value of the aforementioned ratio are indicative of different deformation mechanisms and crack development in the coating and the substrate. A substrate that is made of ductile material (alloy or steel) deforms plastically, whereas a hard coating that is much stiffer than the substrate may not follow the deformation of the substrate and crack brittle.

As mentioned, the improvement of the cavitation erosion resistance depends on the coating properties. In many cases, with increasing coating hardness, the cavitation erosion resistance increases as well (Table 7). However, the test results presented in [113,114] showed the threshold values of the H/E or H^3^/E^2^ ratios at which the best resistance is achieved. These threshold values are about 0.07 and 0.1, respectively. Increasing H/E or H^3^/E^2^ ratios above these values increases the erosion rate. This is because brittleness increases with stiffness and brittle coatings deposited on much ductile substrate are easily damaged under dynamic loads, as described earlier. In addition, in the case of high coating stiffness, the correlation between surface roughness and erosion rate was in opposition to that obtained for ductile materials [55,74,75]. Although the tests confirmed an increase in surface roughness with the time of testing, a higher rate of erosion was recorded for coatings whose roughness was lower [111,114]. The probable reason is a different degradation mechanism than in ductile materials. 

### 4.5. HVOF/HVAF Coating Deposition

High Velocity Oxygen Fuel (HVOF) and High Velocity Air Fuel (HVAF) coatings have a high hardness, good adhesion properties and low porosity compared to coatings obtained by other thermal spray processes. HVOF and HVAF coatings are much thicker than PVD coatings and much better adhered to the substrate. This makes them attractive as protective coatings, including cavitation erosion protection. The thickness of the coatings is from about 100 μm [115] to about 400 μm [116] or even 630 μm [117], but most of them are about 200 µm thick [118,119,120]. A summary of the effect of the deposition of HVOF coating on the cavitation erosion rate and resistance improvement is shown in Table 8. 

**Figure 9:**
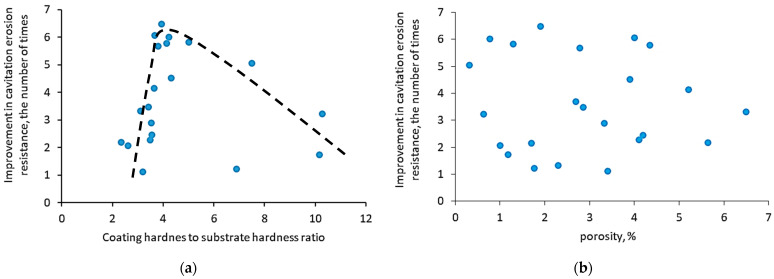
Correlation between hardness ratio, porosity and improvement in erosion resistance of various materials, based on Table 8. (**a**) Effect of the ratio of coating hardness to substrate hardness on the improvement of cavitation erosion resistance; (**b**) Effect of porosity on the improvement on cavitation erosion resistance.

Analysis of the test results showed that the cavitation erosion resistance can be improved by up to 6.5 times (Table 8). This result is lower than that achieved by PVD coatings; however, most of the tested coatings increased the resistance by more than two times, despite porosity which is considered a coating defect. Figure 9 shows the effect of the ratio of coating hardness to substrate hardness on the improvement of cavitation erosion resistance and the effect of porosity on the improvement of this resistance. The greatest increase in resistance improvement was noted for the ratio of coating hardness to substrate hardness of about four (Figure 9a). A further increase in this ratio was accompanied by a decrease in resistance improvement, but it was still greater than 2-fold. A high value of this ratio indicates the different stiffness of the substrate and the coating. Under a dynamic load, the substrate may deform plastically, but the coating may crack brittle. 

Figure 9b shows no correlation between porosity and the improvement in cavitation erosion resistance. This result is surprising and indicates that other properties have a greater impact on the cavitation erosion resistance than overall porosity. No correlation with porosity was also obtained by Hauer et al. who studied the effect of spraying technology (cold spraying, HVOF spraying, warm spray and arc spraying) using N_2_ or H_2_ as the process gas [125]. On the other hand, Sugiyama et al. [126] who tested various WC-CrC-Ni and WC-Co coatings showed a decrease in erosion resistance with the increase in the density of pores. The lack of any relationship could be due to the different pore sizes. Large pores, even in a small amount, cause much more damage than a large number of fine pores, despite the same overall porosity. Unfortunately, it is rarely possible to know the exact distribution of pores, and this information is probably more critical to erosion resistance than the global porosity of the coating or material being tested. 

Szala et al. [115] tested three NiCoCr- based coatings that differed in the content of these elements. With the increase in the hardness of the coating, a decrease in erosion resistance was obtained. The NiCrMoNbTa coating, which had the highest content of Ni (64.56 wt.%) and the lowest hardness (342 HV), had the lowest erosion rate. This result contradicts the results of Sugiyama et al. [126], who showed a decrease in the erosion resistance of the WC-CrC-Ni coatings with the addition of Ni greater than 20%. After the cavitation erosion test, the NiCrMoNbTa coating was characterized by uniform surface roughness and small pitting. The best resistance of this coating was explained by the occurrence of plastic deformation, which slowed down the growth of large craters and the loss of material. In addition, an increase in surface roughness was observed with an increase in mass loss (erosion rate), which is consistent with the study of ductile solid materials [55,74,75]. 

Ding et al. [123] investigated the effect of powder particle size on coating properties and cavitation erosion resistance. A powder size in the range of 20−53 μm was used for the multi-dimensional coating, while the bimodal and nanostructured WC−10Co4Cr coatings were made of powder sizes in the range of 15−45 μm. In the bimodal powder, the volume ratio of WC original crystal of micro-sized (~1.5 μm) to nano-sized (80−180 nm) WC particles was 7:3. In nanostructured WC−10Co4Cr powder, the WC original crystal size is 100−500 nm. The highest porosity was obtained for the bimodal coating and the lowest for the nanostructured coating. The multidimensional coating had the lowest hardness and the nanostructured coating had the highest. It was also shown that the fracture toughness decreased with increasing hardness. Thus, the porosity did not affect the hardness and fracture toughness of the coating. Cavitation erosion studies showed that the rate of erosion decreased with decreasing hardness and increasing fracture toughness, while porosity had no effect. Thus, the effect of coating hardness was similar to that of Szala et al. [115] and the effect of porosity was similar to that shown in Figure 9b. The decrease in the cavitation erosion rate of HVOF coatings with increasing fracture toughness was also achieved in [116,120]. 

A similar impact of fracture toughness of the coatings on the cavitation erosion resistance was obtained by Lamana et al. [124] who tested the effect of the HVOF torches on the properties of WC-Co-based coatings. Coatings produced using a gas fuel torch had lower compressive residual and thermal stresses, as well as a lower hardness but higher fracture toughness and porosity compared to those produced using a liquid fuel torch. With increasing porosity, fracture toughness decreased. Cavitation erosion tests showed that the coatings produced using a liquid fuel torch had less volume loss indicating correlations between erosion resistance and fracture toughness and porosity. The increase in erosion resistance was proportional to the increase in fracture toughness, in contrast to porosity. A 7-fold decrease in porosity caused a 3.5-fold increase in resistance to cavitation erosion. 

Due to marine applications, investigations of nickel aluminum bronzes (NAB; e.g., CuAl10Fe5Ni5), which are used for ship propellers, are important. Hauer et al. investigated the effect of different spraying technology on the cavitation erosion resistance of such a coating deposited on VL-A (S235JR) steel and compared it to bulk NAB [125]. The best result was obtained for the coating applied by cold spraying with N_2_ as the process gas (erosion rate = 0.30 µm/h, coating hardness = 278 HV, porosity = 1.7%). The NAB coating applied with the HVOF method had a more than five times higher erosion rate (1.74 µm/h), despite higher hardness (400 HV) and lower porosity (0.85%). In addition, NAB coating applied with the HVOF method had a higher erosion rate than bulk NAB.

Comparing the coatings produced by both the HVOF and HVAF methods, those produced by the HVAF method had better cavitation erosion resistance [117,127,128,129]. Silveira et al. tested FeCrMnSiB and FeCrMnSiNi coatings deposited on AISI 1020 steel [127]. The HVAF coatings had a higher hardness than the corresponding HVOF coatings. For example, the FeCrMnSiB coating produced using the HVAF method, despite being more porous than the HVOF coating, was characterized by 1.6 times better erosion resistance. In the case of the FeCrMnSiNi coating, the HVAF coating had a 55% lower porosity and two times better resistance. Similar results, i.e., higher hardness, lower porosity and higher erosion resistance of HVAF coatings compared to HVOF coatings, were obtained in [117,128,129]. In addition, some HVOF coatings (e.g., Cr_3_C_2_–25NiCr and Cr_3_C_2_–37WC–18NiCoCr) had a lower resistance than uncoated GX4CrNi13-4 martensitic steel (CA6NM) used as a reference material, unlike HVAF coatings [128,129]. This result, i.e., deterioration of erosion resistance as a result of HVOF coating deposition, is in line with the results of Hauer et al. [125]. 

## 5. Summary

Energy production from renewable sources is currently a very important undertaking. Hydropower plants allow the production of the cheapest energy; however, hydro turbines meet several problems, e.g., degradation of turbine blades caused by erosion processes. In order to protect them, tests were carried out to determine the material resistant to this damage, as well as to select the material properties that reduce the rate of erosion. Tests of solid materials (steel and alloys) have shown that hardness is a key property. However, there are also other properties, such as fracture toughness or ductility (elongation), which also affect erosion resistance.

This review confirmed the beneficial effect of hardness. Nevertheless, it has also been shown that there is not one but several such correlations. The occurrence of several correlations is the result of taking into account different groups of material and of tests being carried out in different conditions (different cavitation intensities). In addition to hardness, the resistance to erosion is also affected by ductility, impact energy, fatigue strength and material structure. The impact of the structure is especially visible when materials with the same or comparable hardness were tested. Hard particles in a ductile matrix that improve wear resistance are detrimental to cavitation erosion resistance in general. However, their shape is also important. Spherical particles are not as harmful as lamellar. Impacts of micro-jets caused the removal or brittle fracture of hard particles as well as plastic deformation in a ductile matrix or ductile grains in the form of pits. This process of surface damage increases surface roughness. Although the increase in roughness with the time of testing is an undeniable fact, the rate of these changes depends on the material being tested. This has led to several models correlating erosion rate with roughness or pitting distribution. The use of a detailed analysis of the shape and number of pits allowed for a very good agreement between the model and experimental results. Unfortunately, this method is very time- and labor-consuming and requires specialized scientific equipment. For this reason, the parameter Ra is more often used in the analysis of the damage and the erosion description. However, many of the obtained dependencies are of very limited use. This is due to the fact that in cavitation erosion, the location of the roughness measurement has a very large impact on the obtained results. The material properties also have a very strong influence on the development of surface roughness. The results and models obtained for ductile materials are not usable for brittle materials.

The results of the tests on solid materials, in which the properties critical for resistance to cavitation erosion were selected, provided guidance on how to protect materials against this type of degradation. As the key property is hardness, to improve the resistance of materials to cavitation erosion, various methods are used to increase the hardness of the material surface. Thermal and thermo-chemical treatment is the oldest method of improvement of material properties, especially surface properties. A combination of carburizing, induction surface hardening, water quenching and annealing allowed a 20 times increase in the resistance to cavitation erosion. Plasma nitriding was also very effective in the reduction of the erosion rate (improving erosion resistance). Depending on the plasma nitriding conditions, the improvement in erosion resistance was from 1.15 times [87] to 23.7 times [86]. In some cases, a deterioration of the protective properties was obtained. This shows how important it is to select the right treatment conditions: process temperature, time and composition of the gas mixture. Because after nitriding the topmost surface layer has some cracks and pores, its removal due to polishing can increase the protection properties. In the case of titanium, such treatment improved the resistance by about eight times. The stellite 250 subjected to low-temperature plasma carbonitriding had a lower resistance than the untreated alloy [90]. A similar effect was obtained for titanium after gas nitriding at 1000 °C [92]. Therefore, when planning heat treatment, it should be borne in mind that it does not always guarantee improved erosion resistance. 

Another method used to increase erosion resistance is to increase the hardness of the surface layer as a result of mechanical action, e.g., by shot peening, deep rolling or friction stir processing. All of these processes introduce compressive stresses into the surface layer, which is considered beneficial. Cavitation erosion tests confirmed the improvement in resistance as a result of such treatment. Nevertheless, the introduced stresses promote corrosion and increase the rate of erosion when the tests are carried out in a corrosive environment, e.g., 3.5 wt.% NaCl solution. This indicates a limitation of the application of this method. 

Laser processing enables remelting or alloying of the surface layer. Both processes allow for an increase in the hardness of the surface and also affect the structure and roughness of the surface layer. The improvement in erosion resistance after applying this treatment was not as spectacular as in the case of plasma nitriding, but in most cases, such an improvement was noted, although it was less than two-fold. It should be noted that thanks to the appropriate selection of conditions of the processing and powders used for alloying the surface, an increase in resistance even by 40 times was also obtained [103]. 

PVD coatings are thin and hard coatings used mainly in tribological applications. Deposition of such coatings improved the resistance to cavitation erosion from 1.3 times [109] up to even 49 times [108]. The best improvement was achieved for the NiCrAlTi coating deposited on 304 steel using direct current magnetron sputtering. However, in the case of using commercially produced TiN coating on Ti6Al4V alloy, a reduction in the resistance was observed [109]. Tests of PVD coatings have shown that for an increase in the ratio of coating hardness to substrate hardness above 3, the improvement in resistance decreases. In addition, there are the threshold values of the H/E or H^3^/E^2^ ratios at which the best resistance is achieved. 

Other types of coating used to improve resistance to cavitation erosion are HVOF and HVAF coatings. These coatings are much thicker than PVD coatings (from about 100 μm to about 400 μm), but this did not result in a greater improvement in resistance compared to PVD coatings. The best improvement (6.5 times) was found for the Cr_3_C_2_–NiCr coating with a hardness of 11.4 GPa and porosity of 1.9% [121]. As in the case of PVD coatings, a threshold value of the ratio of coating hardness to substrate hardness was obtained at which the greatest improvement in resistance to cavitation erosion was obtained. This threshold value was approximately four. Analysis of a significant number of coatings showed no correlation between resistance and porosity. (Fe_3_Al)_30_Ti_35_BN_35_ coating heat-treated at 1000 °C with a porosity of 4% caused a similar improvement in the cavitation erosion resistance (6.1 times) [121] as the Fe-based coating with a hardness of 1099 HV and porosity of 0.77% (6.01 times) [122], and was much better than the Fe-based coating with a hardness of 680 HV and porosity of 1% (2.07 times) [122].

This analysis of the test results has confirmed the effect of hardness and also shows that very high hardness, and especially a large difference with the hardness of the substrate, may result in little or no effect. When selecting the improvement technique, great attention should be paid to the ability of the modified surface or deposited coating to absorb impact energy through the appropriate structure. 

## Figures and Tables

**Table 1:** Micro-jet velocity based on the literature.

Micro-Jet Velocity	Method of Determining	Reference
130–170 m/s	High-speed camera and calculation	[16]
35–120 m/s	High-speed camera	[17]
25–151 m/s	High-speed camera	[18]
200–500 m/s	High-speed camera	[19]
500 m/s	Measurements	[20]
30–48 m/s	High-speed camera	[14]
75–250 m/s	High-speed camera	[21]
400–430 m/s	High-speed camera	[22]
48–175 m/s	High-speed camera	[23]
200–700 m/s	High-speed camera	[24]
755 m/s	Calculation	[25]
877 m/s	Calculation	[15]
>200 m/s	Calculation	[26]

**Table 3:** Cavitation erosion resistance of materials.

Material	Structure	Test Device	Erosion Rate/ Time of Testing	Hardness	Reference
BS 431S29 quenched from 1040 °C and tempered at 200 °C	Martensite	Vibratory	0.094 mm^3^/h 5 h	427 HV	[41]
BS 431S29 quenched from 1040 °C and tempered at 300 °C	Martensite	Vibratory	0.122 mm^3^/h 5 h	406 HV	[41]
BS 431S29 quenched from 1040 °C and tempered at 400 °C	Martensite	Vibratory	0.086 mm^3^/h 5 h	423 HV	[41]
BS 431S29 quenched from 1040 °C and tempered at 500 °C	Martensite	Vibratory	0.208 mm^3^/h 5 h	325 HV	[41]
BS 431S29 quenched from 1040 °C and tempered at 600 °C	Martensite	Vibratory	0.302 mm^3^/h 5 h	262 HV	[41]
DIN 4112 quenched from 1040 °C and tempered at 200 °C	Martensite	Vibratory	0.02 mm^3^/h 5 h	636 HV	[41]
DIN 4112 quenched from 1040 °C and tempered at 300 °C	Martensite	Vibratory	0.038 mm^3^/h 5 h	577 HV	[41]
DIN 4112 quenched from 1040 °C and tempered at 400 °C	Martensite	Vibratory	0.042 mm^3^/h 5 h	531 HV	[41]
DIN 4112 quenched from 1040 °C and tempered at 500 °C	Martensite	Vibratory	0.062 mm^3^/h 5 h	463 HV	[41]
DIN 4112 quenched from 1040 °C and tempered at 600 °C	Martensite	Vibratory	0.112 mm^3^/h 5 h	330 HV	[41]
17-4PH stainless steels	Martensite	Vibratory	1.03 mm^3/^h 1 h	360 HV	[54]
13Cr–5Ni–Mo	b.c.c. martensite	Rotating disk, 45 m/	0.07 mm^3/^h 48 h	450 HV	[38]
13Cr–5Ni–Mo	b.c.c. martensite	Rotating disk, 34 m/	0.009 mm^3^/h 48 h	450 HV	[38]
AISI W1 (0.96 wt.%C, 0.46 wt.% Mn, 0.18wt% Cr, 0.2wt.% Ni, Fe-rest)	Martensite	Vibratory	0.006 mm^3^/h 8 h	540 HV	[55]
AISI 52100 chromium steel	Martensite with spheroidal carbides	Vibratory	0.036 mm^3^/h 8 h	860 HV	[55]
0Cr13Ni6Mo steel Fe-11.9 wt.%Cr-0.36 wt.%Mn-6.1 wt.%Ni	Martensite and retained austenite	Rotating disk, 45 m/	0.115 mm^3/^h for 20 h 0.171 mm^3/^h for 30 h	-	[50]
AISI 1020 low-carbon steel	Tempered martensite with small amounts of ferrite and retained austenite	Vibratory	0.01 mm^3^/h 8 h	350 HV	[55]
AISI 1085 high-carbon steel	bainite and ferrite in a matrix of tempered martensite.	Vibratory	0.0058 mm^3^/h 8 h	865 HV	[55]
0Cr16Ni5Mo steel Fe-15.0 wt.%Cr-0.45 wt.%Mn-5.0 wt.%Ni	Martensite and ferrite	Rotating disk, 45 m/	0.083 mm^3^/h for 20 h 0.116 mm^3^ /h for 30 h	-	[50]
Fe–20 wt.%Mn–6 wt.%Si–7 wt.%Cr-B	71.4% h.c.p. martensite and austenite	Rotating disk, 45 m/	0.019 mm^3^/h 48 h	220 HV	[38]
Fe–20 wt.%Mn–6 wt.%Si– 7 wt.%Cr-B	71.4% h.c.p. martensite and austenite	Rotating disk, 34 m/	0.011 mm^3^/h 48 h	220 HV	[38]
Fe–20 wt.%Mn–6 wt.%Si– 7 wt.%Cr-A	92.7% austenite and h.c.p. martensite	Rotating disk, 45 m/	0.016 mm^3/^h 48 h	208 HV	[38]
Fe–20 wt.%Mn–6 wt.%Si– 7 wt.%Cr-A	92.7% austenite and h.c.p. martensite	Rotating disk, 34 m/	0.01 mm^3^/h 48 h	208 HV	[38]
AISI 304 hot rolled and annealed	Austenite	Vibratory	0.14 mm^3^/h 5 h	194 HV	[41]
AISI 304	Austenite	Vibratory	0.78 mm^3^/h 20 h	177 HV	[56]
AISI 304	Austenite	Vibratory	2.31 mm^3^/h 5 h	246 HV	[53]
AISI 304L	Austenite	Vibratory 20 kHz, 60 µm	0.159 mm^3^/h 5 h	5.34 GPa (510 HV)	[57]
AISI 316 hot rolled and annealed	Austenite	Vibratory	0.35 mm^3^/h 5 h	194 HV	[41]
Ni-based-SFAc	Austenite	Vibratory	0.77 mm^3^/h 5 h	920 HV	[53]
Fe-0.58 wt.%Mn-5.6 wt.%Ni-12.6 wt.%Cr-0.72 wt.%Mo	Austenite	Vibratory 20 kHz, 60 µm	2.1 mm^3^/h 5 h	298 HB (300 HV)	[58]
Fe-13.9 wt.%Mn-0.216 wt.%Ni-17.3 wt.%Cr- 2 wt.%Mo	Austenite	Vibratory 20 kHz, 60 µm	1.31 mm^3^/h 5 h	240 HB (247 HV)	[58]
Fe-13 wt.%Mn-1.9 wt.%Ni- 17 wt.%Cr-1.5 wt.%Mo	Austenite	Vibratory 20 kHz, 60 µm	0.26 mm^3^/h 5 h	211 HB (218 HV)	[58]
18 wt.%Mn18 wt.%Cr 0.5 wt.%N steel	Austenite	Rotating disk, 45 m/s	0.064 mm^3^/h for 20 h	254 HV	[50]
Fe-18.51 wt.%Cr-18.71 wt.%Mn-0.52 wt.%N	Austenite	Rotating disk, 45 m/s	0.047 mm^3^/h for 30 h	254 HV	[50]
Fe–25 wt.%Mn–6 wt.%Si– 7 wt.%Cr	Austenite	Rotating disk, 45 m/	0.096 mm^3^/h 48 h	215 HV	[38]
Fe–21 wt.%Mn–6 wt.%Si– 9 wt.%Cr	Austenite	Rotating disk, 45 m/	0.014 mm^3^/h 48 h	211 HV	[38]
Fe–25 wt.%Mn–6 wt.%Si– 7 wt.%Cr	Austenite	Rotating disk, 34 m/	0.005 mm^3^ /h 48 h	215 HV	[38]
Fe–21 wt.%Mn–6 wt.%Si– 9 wt.%Cr	Austenite	Rotating disk, 34 m/	0.009 mm^3^ /h 48 h	211 HV	[38]
AISI 430 hot rolled	Ferrite	Vibratory	7.44 mm^3^/h 5 h	180 HV	[41]
AISI 409 hot rolled	Ferrite	Vibratory	4.66 mm^3^/h 5 h	129 HV	[41]
3CR12 annealed at 700 °C	Ferrite	Vibratory	2.5 mm^3^/h 5 h	165 HV	[41]
3CR12 annealed at 800 °C	Ferrite	Vibratory	1.422 mm^3^/h 5 h	141 HV	[41]
3CR12 annealed at 900 °C	Ferrite	Vibratory	0.834 mm^3^/h 5 h	167 HV	[41]
2205 duplex steel	Austenite and ferrite	Vibratory 20 kHz, 80 µm	0.115 mm^3^/h 1 h	233 HV	[59]
S235 JR	Ferrite and perlite	Cavitation tunnel, 2.3 m/s	0.176 mm^3^/h 4 h	148.5 HV	[39]
S235 JR	Ferrite and perlite	Cavitation tunnel, 2.3 m/s	0.74 mm^3^/h 4 h	106 HV	[39]
AISI 1045	Ferrite and perlite	Vibratory	1.92 mm^3^/h 2 h	-	[60]
Austempered ductile iron (ADI)	nodular graphite in ausferrite	Vibratory	1.154 mm^3^/h 4 h	313 HV	[61]
Austempered ductile iron (ADI)	nodular graphite in ausferrite	Vibratory	0.718 mm^3^/h 4 h	364 HV	[61]
Austempered ductile iron (ADI)	nodular graphite in ausferrite	Vibratory	0.449 mm^3^/h 4 h	523 HV	[61]
cast iron EN-GJS-400-15, (3.57 wt.% C, 2.51wt.% Si, 0.23 wt.% Mn, Fe = res)	ferrite–perlite matrix graphite nodules with surrounding ferrite areas	Vibratory	12.04 mm^3^/h 165 min	212.8 HV	[62]
cast iron EN-GJS-400-15, (3.57 wt.% C, 2.51wt.% Si, 0.23 wt.% Mn, Fe = res)	ferrite–perlite matrix graphite nodules with surrounding ferrite areas	Vibratory	6.68 mm^3^/h 165 min	178.7 HV	[62]
cast iron EN-GJS-400-15, (3.57 wt.% C, 2.51wt.% Si, 0.23 wt.% Mn, Fe = res)	ferrite–perlite matrix graphite nodules with surrounding ferrite areas	Vibratory	3.67 mm^3^/h 165 min	344.6 HV	[62]
cast iron EN-GJS-400-15, (3.57 wt.% C, 2.51wt.% Si, 0.23 wt.% Mn, Fe = res)	ferrite–perlite matrix graphite nodules with surrounding ferrite areas	Vibratory	2.73 mm^3^/h 165 min	462.2 HV	[62]
NAB alloy (9.39 wt% Al, 4.73 wt% Ni, 4.53 wt% Fe, 1.07 wt% Mn, 0.11 wt.% Zn, Cu -rest)	copper-rich α matrix phase, β phase, intermetallic κi, κii, κiii and κiv phases	Vibratory 20 kHz, 80 µm	0.081 mm^3^/h 1 h	170 HV	[59]
NAB alloy (78.8 wt% Cu, 11.58 wt% Al, 3.98 wt% Ni, 5.12 wt% Fe, 0.43 wt% Zn and 0.09 wt% Mn)	copper-rich α matrix phase, martensitic β′ phase, intermetallic κi, κii, κiii and κiv phases	Vibratory 20 kHz, 45 µm	0.511 mg/h 8 h	228 HV	[63]
NAB alloy (9.24 wt% Al, 4.73 wt% Ni, 4.56 wt% Fe, 1.31 wt% Mn, 0.06 wt.% C, Cu -rest)	fcc copper-rich α matrix, martensitic bcc β’ phases (or retain β phases), κ phases	Vibratory	0.30 mm^3^/h 10 h	-	[64]
Cu-Al alloy (10.23 wt% Al, 4.19 wt% Fe, 0.03 wt% Ni, 0.15 wt% Mn, 0.03 wt.% C, Cu rest)	fcc copper-rich α matrix, β phases, κ phases, Fe(Al, Cu) phases	Vibratory	0.45 mm^3^/h 10 h	-	[64]
Ti-48Al-2Mn-2Nb (at.%)	Titanium γ structure	Vibratory	0.03 mm^3^/h 20 h	225 HV	[56]
Ti-48Al-2Mn-2Nb (at.%) homogenized	Titanium α grains in B2 matrix	Vibratory	0.05 mm^3^/h 20 h	230 HV	[56]
Ti-52Al (at.%) homogenized	Titanium α grains in B2 matrix	Vibratory	0.05 mm^3^/h 20 h	182 HV	[56]
Ti-25Al-10Nb-3V-1Mo (at.%)	Titanium α grains in B2 matrix	Vibratory	0.08 mm^3^/h 20 h	420 HV	[56]
Ti49-Ni51 (wt.%) alloy	Titanium B2 parent phase	Vibratory	0.4 mg/h 5 h	280 HV	[53]
Ti50-Ni50 (wt.%) alloy	Titanium B19′ martensite	Vibratory	0.42 mg/h 5 h	214 HV	[53]
Ti50Ni40Cu10 alloy(wt.%)	Titanium B2′B19′B19′ phase	Vibratory	0.36 mg/h 5 h	181 HV	[53]
2024T351 aluminum alloy (wt%: Cu3.97, Mg1.43, Mn0.625, Si0.5, Al-bal.)	Aluminum phases	Vibratory self-developed device, 20 kHz	0.16 mg/h 3h	155 HV	[65]
AZ31 magnesium alloy (Al 2.5–3.5%, Zn 0.6–1.4%, Si 0–0.3%, Mn 0–0.2%, Mg –balance, wt%)	Magnesium phases	Vibratory	95 µm/h 6 h	49 ± 3 HV	[66]
WC-15Co (wt.%)	Carbides in Co matrix	Vibratory	0.11 mm^3^/h 20 h	1050 HV	[56]
Al_2_O_3_	Ceramic sintered at 1500 °C	jet-impact device	0.031 mm^3^/h, 40 h	17 GPa (1733 HV)	[67]
Al_2_O3/ZrO_2_	Ceramic composite sintered at 1550 °C	jet-impact device	0.0063 mm^3^/h 40 h	17 GPa (1733 HV)	[67]
ZrO_2_	Ceramic sintered at 1550 °C	jet-impact device	0.0425 mm^3^/h 40 h	14 GPa (1428 HV)	[67]
ZrO_2_/WC	Ceramic composite sintered at 1600 °C	jet-impact device	0.0325 mm^3^/h 40 h	17 GPa (1733 HV)	[67]

**Table 4:** Effect of thermo-chemical treatment on erosion rates.

Material /Testing Liquid	Modification	Erosion Rate Time Testing	Hardness	Improvement in Cavitation Erosion Resistance	Reference
16MnCr5 steel	Carburizing in gas at 880 °C, 8 h, and annealing at 180 °C, 90 min	0.16 µm/min 165 min	500–550 HV	2.18 times	[85]
16MnCr5 steel	Carburizing in gas at 880 °C, 8 h, induction hardening using a specific power DP = 0.9 kW cm^–2^, a frequency of 32 kHz, 4 s, followed by water quenching, and annealing at 180 °C, 90 min	0.13 µm/min 165 min	720–780 HV	2.7 times	[85]
Cobalt alloy grade Stellite 6	Nitrogen Ion Implantation 5 × 10^16^ N^+^/cm^−2^	0.0018 mg/min 60 min		2 times	[46]
Cobalt alloy grade Stellite 6	Nitrogen Ion Implantation 1 × 10^17^ N^+^/cm^−2^	0.0022 mg/min 60 min		1.76 times	[46]
ASTM A743 grade CA-6NM steel	Plasma nitriding at 500 °C, 2 h, 532 Pa, gas flow: 5×10^−6^ m^3^ s^−1^, mixture: 5% N_2_ + 95% H_2_ (in volume)	0.034 mg/min 7 h	950 HV	2.58 times	[86]
ASTM A743 grade CA-6NM steel	Plasma nitriding at 500 °C, 2 h, 532 Pa, gas flow 5×10^−6^ m^3^ s^−1^, mixture: 10% N_2_ + 90% H_2_; (in volume)	0.028 mg/min 7 h	1170 HV	3.14 times	[86]
ASTM A743 grade CA-6NM steel	Plasma nitriding at 500 °C, 2 h, 532 Pa, gas flow of 5×10^−6^ m^3^ s^−1^, mixture: 20% N_2_ + 80% H_2_; (in volume)	0.004 mg/min 7 h	1240 HV	23.72 times	[86]
Co30Cr19Fe alloy	Solubilizing at 1200 °C and plasma nitriding at 350 °C, 20 h, 150 Pa, gas mixture: 25% N_2_ + 75% H_2_; (in volume)	0.009 mg/min 60 min	640 HV	1.15 times	[87]
Co30Cr19Fe alloy	Solubilizing at 1200 °C and plasma nitriding at 400 °C, 20 h, 150 Pa, gas mixture: 75% N_2_ + 25% H_2_; (in volume)	0.002 mg/min 60 min	900 HV	5.4 times	[87]
Co30Cr19Fe alloy	Recrystallizing at 1100 °C and plasma nitriding at 400 °C, 20 h, 150 Pa, gas mixture: 75% N_2_ + 25% H_2_; (in volume)	0.002 mg/min 60 min	970 HV	3.05 times	[87]
13-4 CA6NM steel	Plasma nitriding at 400 °C, 20 h, gas mixture: 25% N_2_ + 75% H^2^.	0.005 µm/min 22 h	1059 HV	3.62 times	[88]
AISI 1045 steel	Plasma nitriding at 430–450 °C, 2 h	0.04 µg/min	4.1 GPa	3.16 times	[89]
13-4 CA6NM steel	Salt bath nitrocarburizing	0.03 µm/min 22 h	1064 HV	22.33 times	[88]
Stellite 250 alloy (dual phased Co–Cr alloy)	Low-temperature plasma carbonitriding at 380 °C, 3 h	0.002 µm/min 15 h	938 HV	1.74 times	[90]
Stellite 250 alloy (dual phased Co–Cr alloy)	Low-temperature plasma carbonitriding at 380 °C, 9 h	0.001 µm/min 15 h	898 HV	2.79 times	[90]
Stellite 250 alloy (dual phased Co–Cr alloy)	Low-temperature plasma carbonitriding at 380 °C, 15 h.	0.006 µm/min 15 h	997 HV	0.59 times	[90]
Titanium (CP-Ti)	Gas nitriding at 700 °C, 16 h	0.0089 mg/min 8 h	1023.5 HV	2.53 times	[91]
Titanium (CP-Ti)	Gas nitriding at 850 °C, 4 h	0.0062 mg/min 8 h	977.4 HV	3.63 times	[91]
Titanium (CP-Ti)	Gas nitriding at 850 °C, 8 h	0.0157 mg/min 8 h	1083.7 HV	1.42 times	[91]
Titanium (CP-Ti)	Gas nitriding at 850 °C, 16 h	0.0187 mg/min 8 h	1158.6 HV	1.2 times	[91]
Titanium (CP-Ti)	Gas nitriding at 1000 °C, 16 h	0.0304 mg/min 8 h	1225.3 HV	0.74 times	[91]
Ti6Al4V alloy	Laser gas nitriding using the scanning galvanometer	0.038 mg/min 13 h	730 HV	2.5 times	[92]
Ti6Al4V alloy	Gas nitriding using the diode laser (laser spot size d = 4 mm) with the robot	0.05 mg/min 13 h	1000 HV	1.89 times	[92]
ASTM A743 steel (CA6NM)	Boronised using the packing method at 950 °C for 2 h	0.03 µm/min 15 h	1950 HV	2.9 times	[93]
ASTM A743 steel (CA6NM)	Boronised using the packing method at 950 °C for 6 h	0.048 µm/min 15 h	1967 HV	1.82 times	[93]
ASTM A743 steel (CA6NM)	Boronised using the packing method at 950 °C for 8 h	0.049 µm/min 15 h	1964 HV	1.77 times	[93]

**Table 5:** Effect of mechanical treatment on erosion rates.

Material /Testing Liquid	Modification	Erosion Rate Test Duration	Hardness (Residual Stress)	Improvement in Erosion Resistance	Reference
2024T351 Al alloy Cu3.97, Mg1.43, Mn0.625, Si0.5 and Al-bal, wt% water	Ultrasonic shot peening with vibration intensity of 60%, duration of 60 s	0.0015 mg/min 3 h	170 HV (−185.3 MPa)	1.76 times	[65]
2024T351 Al alloy Cu3.97, Mg1.43, Mn0.625, Si0.5 and Al-bal, wt% water	Ultrasonic shot peening with vibration intensity of 80%, duration of 60 s	0.0012 mg/min 3 h	175 HV (−263.6 MPa)	2.14 times	[65]
2024T351 Al alloy Cu3.97, Mg1.43, Mn0.625, Si0.5 and Al-bal, wt% water:	Ultrasonic shot peening with vibration intensity of 80%, duration of 120 s	0.002 mg/min 3 h	182 HV (−283.5 MPa)	1.3 times	[65]
2024T351 Al alloy Cu3.97, Mg1.43, Mn0.625, Si0.5 and Al-bal, wt% water	Ultrasonic shot peening with vibration intensity of 80%, duration of 240 s	0.0024 mg/min 3 h	192 HV (−332.1 MPa)	1.12 times	[65]
304 steel water	Deep rolling	0.006 mm^3^/min 6 h	325 HV	2.23 times	[94]
316 steel water	Deep rolling	0.0096 mm^3^/min 6 h	270 HV	2.18 times	[94]
316 steel water	friction stir processing at 388 rpm	0.00046 mm^3^/min 20 h	420 HV	5.1 times	[95]
316 steel water	friction stir processing at 1800 rpm	0.0006 mm^3^/min 20 h	350 HV	3.8 times	[95]
316 steel 3.5% NaCl	friction stir processing at 388 rpm	0.0007 mm^3^/min 19 h	420 HV	4.46 times	[95]
316 steel 3.5% NaCl	friction stir processing at 1800 rpm	0.001 mm^3^/min 19 h	350 HV	3.38 times	[95]
nickel-aluminum bronze (NAB) alloy distilled water	Compressive stresses introduced; −60 MPa	0.009 mg/cm^2^ min 8 h	−60 MPa	0.95 times	[63]
nickel-aluminum bronze alloy distilled water	Compressive stresses introduced; stress level: −120 MPa	0.0092 mg/cm^2^ min 8 h	−120 MPa	0.92 times	[63]
nickel-aluminum bronze (NAB) alloy 3.5% NaCl	Compressive stresses introduced; stress level: −60 MPa	0.016 mg/cm^2^ min 8 h	−60 MPa	0.8 times	[63]
nickel-aluminum bronze (NAB) alloy 3.5% NaCl	Compressive stresses introduced; stress level: −120 MPa	0.022 mg/cm^2^ min 8 h	−120 MPa	0.57 times	[63]

**Table 6:** Laser processing effect.

Substrate Material /Testing Liquid	Modification	Erosion Rate	Hardness	Improvement in Erosion Resistance	Reference
IRECA alloy/water	200 W, 20 mm/s, position +20 mm	0.35 mg/min 9 h	38–39 HRC	1.39 times	[96]
IRECA alloy/water	210 W, 10 mm/s, position +0 mm	0.32 mg/min 9 h	39–42 HRC	1.49 times	[96]
IRECA alloy/water	1 pass: 210 W, 20 mm/s, position +0 mm and 2 pass: 150 W, 5 mm/s, position 0 mm	0.3 mg/min 9 h	36–38 HRC	1.58 times	[96]
IRECA alloy/water	300 W, 10 mm/s, position 0 mm	0.33 mg/min 9 h	36–37 HRC	1.45 times	[96]
IRECA alloy/water	210 W, 10 mm/s, position 0 mm	0.42 mg/min 9 h	41–43 HRC	1.15 times	[96]
IRECA alloy/water	275 W, 10 mm/s, position 0 mm	0.29 mg/min 9 h	34–36 HRC	1.67 times	[96]
AA6061 aluminum alloy/water	Laser melting	0.125 mg/min cm^2^ 4 h	59 HV	0.99 times	[97]
AA6061 aluminum alloy/water	Laser surface alloying 100% SiC + 0% Si_3_N_4_	0.11 mg/min cm^2^ 4 h	377 HV	1.13 times	[97]
AA6061 aluminum alloy/water	Laser surface alloying 95% SiC + 5% Si_3_N_4_	0.094 mg/min cm^2^ 4 h	386 HV	1.32 times	[97]
AA6061 aluminum alloy/water	Laser surface alloying 80% SiC + 20% Si_3_N_4_	0.087 mg/min cm^2^ 4 h	353 HV	1.43 times	[97]
AA6061 aluminum alloy/water	Laser surface alloying 50% SiC + 50% Si_3_N_4_	0.061 mg/min cm^2^ 4 h	378 HV	2.04 times	[97]
AA6061 aluminum alloy/water	Laser surface alloying 0% SiC + 100% Si_3_N_4_	0.041 mg/min cm^2^ 4 h	400 HV	3.02 times	[97]
17-4PH stainless steel/water	Laser surface alloying Stellite 6 powder (powder size 45~150 μm)	0.041 mg/min 50 h	461 HV	5 times	[54]
17-4PH stainless steel/water	Laser surface alloying Wrought Stellite 6B powder	0.03 mg/min 50 h	430 HV	7 times	[54]
17-4PH stainless steel/water	Laser surface alloying low-carbon high-molybdenum C14 powder (powder size 53~180 μm)	0.017 mg/min 50 h	487 HV	12 times	[54]
304 steel/water	laser shock processing	0.028 mg/min 6 h		3 times	[98]
AA5083 aluminium alloy/water	laser shock peening with an ablative coating (LSP)	0.588 mg/min 300 min	108 HV −175 MPa	1.45 times	[99]
AA5083 aluminium alloy/water	laser shock peening without ablative coating (LSPwC)	0.399 mg/min 300 min	119 HV −189MPa	2.13 times	[99]
AISI 420 stainless/ water	laser shock peening with one coverage layer	0.011 mg/min 10 h	310 HV −387 MPa	1.5 times	[100]
AISI 420 stainless/ water	laser shock peening with two coverage layers	0.009 mg/min 10 h	330 HV −400 MPa	1.8 times	[100]

**Table 7:** PVD coating deposition effect.

Coating Substrate Material	Modification	Erosion Rate /Time Testing	Coating Hardness /Substrate Hardness	Improvement in Erosion Resistance	Reference
Cr_1-x_N_x_ coating AISI 1045 carbon steel	Plasma-Assisted PVD	0.0625 mg/min 8 h	4.05 GPa / 3 GPa	2 times	[89]
Cr_1-x_N_x_ coating AISI 1045 carbon steel	Plasma nitride substrate surface and Plasma-Assisted PVD	0.0167 mg/min 8 h	5.76 GPa / 3 GPa	7.5 times	[89]
Cr-N coating X39Cr13 steel hardening and annealing at 600 °C	Cathodic arc evaporation method	0.001 mg/min 10 h	19.2 GPa / 2.8 GPa	1.5 times	[105]
Cr-N coating X39Cr13 steel hardening and annealing at 600 °C	Cathodic arc evaporation method	0.0067 mg/min 10 h	21.4 GPa / 2.8 GPa	2.75 times	[105]
TiN coating X39Cr13 steel hardening and annealing at 600 °C	Cathodic arc evaporation method	0.0095 mg/min 10 h	25.4 GPa / 4,5 GPa	1.58 times	[105]
TiN coating X39Cr13 steel hardening and annealing at 400 °C	Cathodic arc evaporation method	0.0075 mg/min 10 h	27.4 GPa / 4.5 GPa	2.4 times	[105]
TiN coating Ti6Al4V substrate	Commercially produced	0.067 mg/min 30 min	2200 HK / 405 HK	0.2 times	[106]
AlTiN coating AISI 304 steel	DC magnetron sputtering	-	35.9 GPa	11 times	[107]
TiAlN coating AISI 304 steel	DC magnetron sputtering	-	32.6 GPa	6.6 times	[107]
NiCrAlTi coating 304 L steel	Direct current magnetron sputtering (DCMS)	0.00005 mm^3^/min 10 h	713 HV / 217 HV	49 times	[108]
NiCrAlTi-1N coating 304 L steel	Direct current magnetron sputtering (DCMS) gas mixtures with Ar flux of 8 sccm and N_2_ flux of 1 sccm	0.0001 mm^3^/min 10 h	641 HV / 217 HV	25 times	[108]
NiCrAlTi-3N coating 304 L stainless steel	Direct current magnetron sputtering (DCMS) gas mixtures with Ar flux of 8 sccm and N_2_ flux of 3 sccm	0.0002 mm^3^/min 10 h	770 HV / 217 HV	11 times	[108]
NiCrAlTi-5N coating 304 L stainless steel	Direct current magnetron sputtering (DCMS) gas mixtures with Ar flux of 8 sccm and N_2_ flux of 5 sccm	0.0007 mm^3^/min 10 h	677 HV / 217 HV	3.3 times	[108]
NiCrAlTi-8N coating 304 L stainless steel	Direct current magnetron sputtering (DCMS) gas mixtures with Ar flux of 8 sccm and N_2_ flux of 8 sccm	0.0014 mm^3^/min 10 h	568 HV / 217 HV	1.8 times	[108]
NiTi/TiCN, coating Thickness ratio 2:1 X38CrMoV51 steel	Magnetron sputtering	0.009 mg/min 12 h	7 GPa / 3 GPa	1.5 times	[109]
NiTi/TiCN, coating Thickness ratio 1:1 X38CrMoV51 steel	Magnetron sputtering	0.007 mg/min 12 h	7.2 GPa / 3 GPa	1.9 times	[109]
NiTi/TiCN, coating Thickness ratio 1:2 X38CrMoV51 steel	Magnetron sputtering	0.005 mg/min 12 h	7.36 GPa / 3 GPa	2.8 times	[109]
CrAlYN/CrN Thickness ratio 1:1 Ti6AlV substrate	High Power Impulse Magnetron Sputtering (HIPIMS) with high ion bombarding energy	0.0099 mg/min 4,5 h	3000 HK / 405 HK	1.3 times	[106]
CrAlYN/CrN Thickness ratio 1:1 Ti6AlV substrate	High Power Impulse Magnetron Sputtering (HIPIMS) with low ion bombarding energy	0.0008 mg/min 4.5 h	2700 HK / 405 HK	15.5 times	[106]

**Table 8:** Effect of deposition of HVOF coating.

Coating Substrate Material	Erosion Rate /Time Testing	Porosity	Coating Hardness /Substrate Hardness	Improvement in Erosion Resistance	Reference
(Fe_3_Al)_30_Ti_35_BN_35_ AISI 444 steel	0.073 mg/min 500 min	3.9%	12.5 GPa 2.9 GPa	4.5 times	[121]
(Fe_3_Al)_30_Ti_35_BN_35_ AISI 444 steel heat-treated at 1000 °C	0.054 mg/min 500 min	4%	10.6 GPa 2.9 GPa	6.1 times	[121]
(Fe_3_Al)_30_Ti_35_BN_35_ AISI 444 steel heat-treated at 1400 °C	0.099 mg/min 500 min	6.5%	9 GPa 2.9 GPa	3.3 times	[121]
WC–CoCr AISI 444 steel	0.056 mg/min 500 min	1.3%	14.5 GPa 2.9 GPa	5.8 times	[121]
Cr_3_C_2_–NiCr AISI 444 steel	0.051 mg/min 500 min	1.9%	11.4 GPa 2.9 GPa	6.5 times	[121]
WC-NiCr coating Monel K-500	0.0004 mm^3^/min 10 h	4.2%	1406 HV 395 HV	2.5 times	[120]
WC-18Hastelloy C Monel K-500	0.0009 mm^3^/min 10 h	3.4%	1261 HV 395 HV	1.1 times	[120]
Fe-based coating 321 steel	0.012821 mg/min 27 h	3.33%	920 HV 260 HV	2.90 times	[122]
Fe-based coating 321 steel	0.010684 mg/min 27 h	2.86%	890 HV 260 HV	3.47 times	[122]
Fe-based coating 321 steel	0.008961 mg/min 27 h	5.21%	950 HV 260 HV	4.14 times	[122]
Fe-based coating 321 steel	0.017921 mg/min 27 h	1.0%	680 HV 260 HV	2.07 times	[122]
Fe-based coating 321 steel	0.01634 mg/min 27 h	4.1%	908 HV 260 HV	2.27 times	[122]
Fe-based coating 321 steel	0.006173 mg/min 27 h	0.77%	1099 HV 260 HV	6.01 times	[122]
Fe-based coating 321 steel	0.017007 mg/min 27 h	5.64%	610 HV 260 HV	2.18 times	[122]
Fe-based coating 321 steel	0.006536 mg/min 27 h	2.78%	987 HV 260 HV	5.68 times	[122]
Fe-based coating 321 steel	0.00641 mg/min 27 h	4.35%	1073 HV 260 HV	5.79 times	[122]
WC-Co-Cr AZ31 magnesium alloy	0.145 mg/min 6 h	2.7%	15.12 GPa	3.7 times	[66]
WC-Co AZ31 magnesium alloy	0.405 mg/min 6 h	2.3%	16.84 GPa	1.32 times	[66]
WC-Cr_3_C_2_-Ni AZ31 magnesium alloy	0.248 mg/min 6h	1.7%	13.15 GPa	2.15 times	[66]
WC-12Co coating 304 steel	0.0072 mm^3^/min 16 h	0.63%	1541 HV 150 HV	3.23 times	[116]
WC-12Co coating 304 steel	0.0134 mm^3^/min 16 h	1.18%	1523 150 HV	1.73 times	[116]
WC-12Co coating 304 steel	0.019 mm^3^/min 16 h	1.76%	1034 150 HV	1.22 times	[116]
WC-10Co4Cr coating 304 steel	0.00199 mm^3^/min 12 h	0.31%	1126 150 HV	5.06 times	[123]
WC-10Co4Cr coating 304 steel	0.00305 mm^3^/min 12 h	0.47%	1186 150 HV	3.29 times	[123]
WC-10Co4Cr coating 304 steel	0.00278 mm^3^/min 12 h	0 26%	1241 150 HV	3.62 times	[123]
WC-12Co GF AISI 1008 steel	0.0466 mm^3^/min 128 min	3.32%	860 HV 100 HV		[124]
WC-12Co GF AISI 1008 steel	0.0212 mm^3^/min 128 min	1.3%	1069 HV 100 HV		[124]
WC-12Co GF AISI 1008 steel	0.257 mm^3^/min 128 min	3.15%	890 HV 100 HV		[124]
WC-12Co GF AISI 1008 steel	0.0124 mm^3^/min 128 min	0.47%	989 HV 100 HV		[124]
CuAl9Ni5-Fe4Mn (NAB) VL-A steel (S235JR	0.029 mm/min 120 min	0.85%	400 HV	0.23 times	[125]

## Data Availability

The data presented in this study are openly available in the publications cited.
